# A Facile and Mild Synthesis of Trisubstituted Allylic Sulfones from Morita-Baylis-Hillman Carbonates

**DOI:** 10.3390/molecules20058213

**Published:** 2015-05-07

**Authors:** Lin Jiang, Yong-Gen Li, Jiang-Feng Zhou, Yong-Ming Chuan, Hong-Li Li, Ming-Long Yuan

**Affiliations:** 1 Engineering Research Center of Biopolymer Functional Materials of Yunnan, School of Chemistry and Biotechnology, Yunnan Minzu University, Kunming 650500, China; E-Mails: lyg1990wsh@163.com (Y.-G.L.); zjf109lq@163.com (J.-F.Z.); chuan5211017@126.com (Y.-M.C.); honglili_1982@163.com (H.-L.L.); 2 Key Laboratory of Chemistry in Ethnic Medicinal Resources, State Ethnic Affairs Commission & Ministry of Education, School of Chemistry and Biotechnology, Yunnan Minzu University, Kunming 650500, China

**Keywords:** Morita-Baylis-Hillman carbonate, allylic sulfone, trisubstituted alkene, allylic substitution

## Abstract

An efficient and catalyst-free synthesis of trisubstituted allylic sulfones through an allylic sulfonylation reaction of Morita-Baylis-Hillman (MBH) carbonates with sodium sulfinates has been developed. Under the optimized reaction conditions, a series of trisubstituted allylic sulfones were rapidly prepared in good to excellent yields (71%–99%) with good to high selectivity (*Z/E* from 79:21 to >99:1). Compared with known synthetic methods, the current protocol features mild reaction temperature, high efficiency and easily available reagents.

## 1. Introduction

**Scheme 1:**
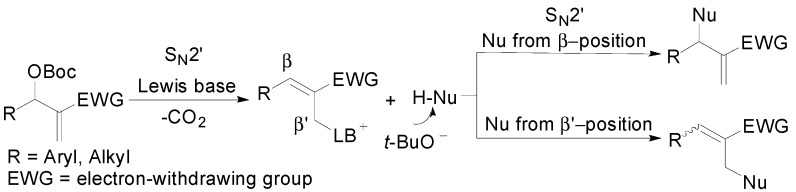
Allylic substitution reaction of Morita-Baylis-Hillman (MBH) carbonates.

Morita-Baylis-Hillman (MBH) adducts and their derivatives are very useful multifunctional synthons in organic chemistry [1,2,3,4,5]. Since the pioneering work of Lu and co-workers in 2004, MBH carbonates have triggered much interest among chemistry researchers [6]. The most extensively studied transformation pattern of this type of MBH derivatives is the allylic substitution with a pronucleophile in the presence of a Lewis basic catalyst [7,8,9]. Based on the substitution position on MBH carbonates, the transformations could be divided into the following two styles: substitution at the β-position through a S_N_2'-S_N_2' cascade or substitution at the β'-position via a single S_N_2' route (Scheme 1). Compared with the former route, which is widely employed in asymmetric synthesis to access versatile multifunctional chiral molecules [10,11,12,13,14,15,16], the latter route has been often used for the preparation of trisubstituted alkenes [17,18,19,20].

**Scheme 2:**
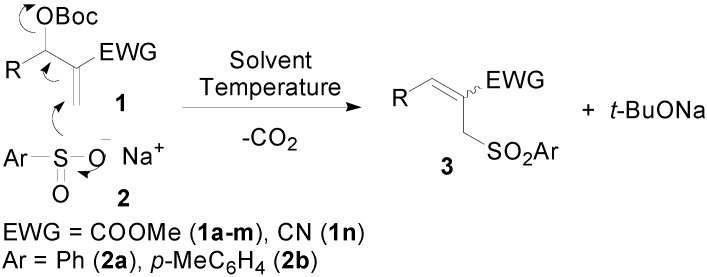
Allylic sulfonylation of MBH carbonates **1** with sodium sulfinates **2**.

Allylic sulfone derivatives are important intermediates in organic synthesis [21,22,23,24]. Recent studies have revealed that these compounds exhibit remarkable biological activities [25]. The use of MBH adducts or their acetates as good starting materials for the trisubstituted allylic sulfones has been reported in some instances [26,27,28,29,30,31,32,33,34]. Although many sulfonyl precursors including sulfinate [26,27,28,29], *p*-toluenesulfonylmethylcyanide [30], arenesulfonyl cyanide [31], sulfinyl chloride [32], sulfonylhydrazide [33] and sulfinic acid [34] have been employed in this type of allylic sulfonylation, sulfinate is undoubtedly a cheap and easily available reagent. However, either a high reaction temperature [26,27] (70–80 °C, 6–16 h) or unconventional organic solvent (ionic liquids or polyethylene glycol) [28,29], accompanied with tedious work-up procedures, were required to ensure a high yield of the desired products. Since MBH carbonates usually exhibit much superior reactivity to MBH acetates, we envisaged that they would be more susceptible to the nucleophilic attack by sulfinate. Herein, we report a new protocol in which MBH carbonates **1** and sodium sulfinates **2** undergo a smooth and rapid S_N_2' pathway to realize the trisubstituted allylic sulfones **3** under mild and catalyst-free reaction conditions (Scheme 2).

## 2. Results and Discussion

### 2.1. Optimization Studies

Preliminary studies were carried out by using MBH carbonate **1a** (R = Ph, EWG = CO_2_Me) and sodium benzenesulfinate (**2a**, Ar = Ph). The screening results are presented in Table 1. Firstly, the model reaction was investigated with different solvents at ambient temperature (Table 1, entries 1–6). Among the solvents tested, toluene and chloroform gave poor conversion (Table 1, entries 1 and 2), and PhCF_3_ afforded only a trace amount of the final adduct **3a** after 72 h (Table 1, entry 3). Although 1,2-dichloroethane (DCE) and tetrahydrofuran (THF) afforded **3a** in high yield (Table 1, entries 4 and 5), acetonitrile was a superior solvent with regard to both conversion rate and product yield (92%, Table 1, entry 6). Next, it was found that when the reaction temperature was raised to 40 °C, nearly full conversion could be achieved within a significantly shortened reaction time and the expected product was furnished in 96% yield (Table 1, entry 7). Finally, the examination of the reaction with a decreased concentration of substrate **1a** revealed no influence on product yield, whereas the reaction time was prolonged (Table 1, entry 8).

**Table 1:** Optimization of reaction conditions using MBH carbonate **1a** and sodium benzenesulfinate **2a** ^a^.

Entry	Solvent	T (°C)	Time (h)	Yield (%) ^b,c^
1	toluene	25	36	37
2	CHCl_3_	25	48	17
3	PhCF_3_	25	72	trace
4	DCE	25	36	82
5	THF	25	36	87
6	MeCN	25	36	92
**7**	**MeCN**	**40**	**2**	**96 ^d^**
8 ^e^	MeCN	40	5	96

^a^ Reaction conditions: Unless otherwise noted, reactions were performed with **1a** (0.1 mmol) and sodium benzenesulfinate **2a** (0.15 mmol) in solvent (1 mL) at indicated temperature; ^b^ Isolated yield of two inseparable isomers; ^c^ Major isomer of **3a** was determined to be *Z* by comparison of its NMR data with the one reported in literature [35]; ^d^ *Z/E* = 96:4, determined by ^1^H-NMR analysis; ^e^ 2 mL of solvent was used.

### 2.2. Synthesis of Trisubstituted Allylic Sulfones ***3a–o***

On the basis of the above optimized reaction parameters (0.1 mmol of MBH carbonate **1a** and 0.15 mmol of sodium benzenesulfinate (**2a**) to perform the reaction in 1 mL of MeCN at 40 °C), this protocol was then extended to other MBH carbonates or sulfinates to investigate the scope and limitation of the method. As shown in Table 2, MBH carbonates **1** could generally be converted within 2 h and corresponding products **3** were obtained in good to excellent yields (71%–99%) with good to high selectivity (*Z/E* from 79:21 to >99:1) (Table 2, entries 1–15). Different substituents on the phenyl group were first explored. The results showed that the electronic nature or position of substituents had minimal influence on reaction efficiency in terms of reaction rate and product yield in general (78%–98%, Table 2, entries 1–9). Besides, 1-naphthyl group-substituted MBH carbonate **1j** was a suitable substrate, albeit with lower yield (71%, Table 2, entry 10). Meanwhile, two heteroaromatic substrates **1k** and **1l** also showed high reactivity, providing **3k** and **3l** in high yields (85% and 96%, Table 2, entries 11 and 12). It is worth mentioning that the MBH carbonate **1m**, which was prepared from an aliphatic aldehyde, could participate in this reaction to produce the desired product **3m** in high yield (91%, Table 2, entry 13). In addition, MBH carbonate **1n**, which was derived from acrylonitrile, could also be transformed in excellent yield under the catalyst-free reaction conditions (99%, Table 2, entry 14). To our delight, sodium *p*-toluenesulfinate **2b** (Ar = *p*-MeC_6_H_4_) was well tolerated and the desired product **3o** was provided in 95% yield (Table 2, entry 15).

**Table 2:** Substrate scope for allylic sulfonylation of MBH carbonates **1** with sodium sulfinates **2** ^a^.

Entry	R	EWG	Ar	Yield (%) ^b^	*Z/E* ^c,d^
1	Ph (**1a**)	CO_2_Me	Ph	96 (**3a**)	96:4
2	*o*-ClC_6_H_4_ (**1b**)	CO_2_Me	Ph	88 (**3b**)	95:5
3	*p*-ClC_6_H_4_ (**1c**)	CO_2_Me	Ph	96 (**3c**)	85:15
4	*p*-NO_2_C_6_H_4_ (**1d**)	CO_2_Me	Ph	83 (**3d**)	79:21
5	*m*-BrC_6_H_4_ (**1e**)	CO_2_Me	Ph	93 (**3e**)	94:6
6	*p*-MeOC_6_H_4_ (**1f**)	CO_2_Me	Ph	78 (**3f**)	88:12
7	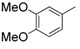 (**1g**)	CO_2_Me	Ph	98 (**3g**)	90:10
8	*m*-MeC_6_H_4_ (**1h**)	CO_2_Me	Ph	96 (**3h**)	88:12
9	*p*-MeC_6_H_4_ (**1i**)	CO_2_Me	Ph	92 (**3i**)	96:4
10	1-naphthyl (**1j**)	CO_2_Me	Ph	71 (**3j**)	96:4
11	2-furyl (**1k**)	CO_2_Me	Ph	85 (**3k**)	>99:1
12	2-thienyl (**1l**)	CO_2_Me	Ph	96 (**3l**)	81:19
13	*n*-propyl (**1m**)	CO_2_Me	Ph	91 (**3m**)	82:18
14	Ph (**1n**)	CN	Ph	99 (**3n**)	<1:99
15	Ph (**1a**)	CO_2_Me	*p*-MeC_6_H_4_	95 (**3o**)	84:16

^a^ Reaction conditions: Unless otherwise noted, reactions were performed with MBH carbonate **1** (0.1 mmol) and sodium sulfinate **2** (0.15 mmol) in MeCN (1 mL) at 40 °C for 2 h; ^b^ Isolated yield of two inseparable isomers; ^c^ Olefin geometry was assigned by analogy with that of **3a**; ^d^ *Z/E* ratio was determined by ^1^H-NMR analysis.

## 3. Experimental Section

### 3.1. General Information

TLC was performed on glass-backed silica plates. Flash column chromatography was performed using silica gel (200–300 mesh) eluting with ethyl acetate and petroleum ether. UV light was used to visualize products. ^1^H-NMR spectra were recorded at 400 MHz, and ^13^C-NMR spectra were recorded at 100 MHz (Avance 400, Bruker, Faellanden, Switzerland). Tetramethylsilane was used as the internal standard. Chemical shifts are reported in ppm downfield from the solvent signal (CDCl_3_, δ = 7.27 ppm) for ^1^H-NMR and relative to the central CDCl_3_ resonance (δ = 77.0 ppm) for ^13^C-NMR spectroscopy. Coupling constants are given in Hz. ESI-HRMS was recorded on a Waters SYNAPT G2 (Milford, MA, USA). In experiments requiring dry solvents, DCE, chloroform and toluene were distilled from CaH_2_. PhCF_3_ was stored over 4 Å molecular sieves. THF was dried over sodium metal. Acetonitrile was dried over P_2_O_5_. All other chemicals were used without purification as commercially available. MBH carbonates were prepared by the reported procedure [36].

### 3.2. General Procedure for Preparation of Trisubstituted Allylic Sulfones ***3a–o***

MBH carbonate **1** (0.1 mmol) and sodium sulfinate **2** (0.15 mmol) were mixed in MeCN (1 mL) and heated at 40 °C for 2 h. Then, the reaction mixture was concentrated under reduced pressure and the residue was diluted with toluene and purified by flash column chromatography on silica gel (petroleum ether/EtOAc) to afford the desired product **3a–o**. Products **3a** [33], **3d** [26], **3f** [26], **3k** [26], **3l** [27], **3n** [33] and **3o** [26] are known compounds.

*(Z)-Methyl 3-phenyl-2-[(phenylsulfonyl)methyl]acrylate* (**3a**). Colourless liquid; 96% yield; *Z/E* = 96:4; ^1^H-NMR: δ = 7.95 (s, 1H), 7.86 (d, *J* = 8.0 Hz, 2H), 7.63–7.59 (m, 1H), 7.52–7.47 (m, 4H), 7.39–7.37 (m, 3H), 4.49 (s, 2H), 3.60 (s, 3H) ppm; ^13^C-NMR: δ = 167.0, 146.6, 139.6, 133.9, 133.8, 129.9, 129.4, 129.2, 129.0, 128.7, 121.1, 55.3, 52.5 ppm; ESI-HRMS: calcd. For C_17_H_16_O_4_S+Na 339.0667, found 339.0661.

*(Z)-Methyl 3-(2-chlorophenyl)-2-[(phenylsulfonyl)methyl]acrylate* (**3b**). Colourless liquid; 88% yield; *Z/E* = 95:5; ^1^H-NMR: δ = 7.94 (m, 1H), 7.75 (d, *J* = 8.0 Hz, 2H), 7.55–7.53 (m, 2H), 7.43–7.40 (m, 2H), 7.30–7.27 (m, 1H), 7.23–7.21 (m, 2H), 4.31 (s, 2H), 3.55 (s, 3H) ppm; ^13^C-NMR: δ = 165.5, 142.4, 138.5, 133.3, 133.0, 131.5, 129.9, 129.2, 129.0, 128.3, 128.1, 127.7, 126.3, 122.3, 54.2, 51.8 ppm; ESI-HRMS: calcd. For C_17_H_15_ClO_4_S+Na 373.0277, found 373.0273.

*(Z)-Methyl 3-(4-chlorophenyl)-2-[(phenylsulfonyl)methyl]acrylate* (**3c**). Colourless liquid; 96% yield; *Z/E* = 85:15; ^1^H-NMR: δ = 7.89 (s, 1H), 7.85 (d, *J* = 8.0 Hz, 2H), 7.64–7.61 (m, 1H), 7.53–7.49 (m, 2H), 7.46 (d, *J* = 8.0 Hz, 2H), 7.35 (d, *J* = 8.0 Hz, 2H), 4.44 (s, 2H), 3.57 (s, 3H) ppm; ^13^C-NMR: δ = 165.6, 144.0, 138.3, 134.9, 132.9, 131.1, 129.6, 128.1, 127.5, 120.4, 54.1, 51.5 ppm; ESI-HRMS: calcd. For C_17_H_15_ClO_4_S+H 351.0458, found 351.0459.

*(Z)-Methyl 3-(4-nitrophenyl)-2-[(phenylsulfonyl)methyl]acrylate* (**3d**). Pale yellow solid; 83% yield; *Z/E* = 79:21; ^1^H-NMR: δ = 8.24 (d, *J* = 8.0 Hz, 2H), 7.98 (s, 1H), 7.86 (d, *J* = 8.0 Hz, 2H), 7.69–7.64 (m, 3H), 7.55–7.52 (m, 2H), 4.40 (s, 2H), 3.63 (s, 3H) ppm; ^13^C-NMR: δ = 165.0, 142.5, 139.0, 133.1, 128.8, 128.2, 127.7, 127.5, 123.2, 122.9, 122.4, 53.9, 51.7 ppm; ESI-HRMS: calcd. For C_17_H_15_NO_6_S+Na 384.0518, found 384.0518.

*(Z)-Methyl 3-(3-bromophenyl)-2-[(phenylsulfonyl)methyl]acrylate* (**3e**). Pale yellow solid; 93% yield; *Z/E* = 94:6; ^1^H-NMR: δ = 7.85–7.83 (m, 3H), 7.68–7.64 (m, 1H), 7.53–7.42 (m, 5H), 7.28–7.24 (m, 1H), 4.46 (s, 2H), 3.67 (s, 3H) ppm; ^13^C-NMR: δ = 166.6, 144.6, 139.1, 135.7, 134.0, 132.6, 131.9, 130.4, 129.3, 128.7, 127.4, 123.0, 122.7, 55.0, 52.7 ppm; ESI-HRMS: calcd. For C_17_H_15_BrO_4_S+Na 416.9772, found 416.9772.

*(Z)-Methyl 3-(4-methoxyphenyl)-2-[(phenylsulfonyl)methyl]acrylate* (**3f**)*.* Colourless liquid; 78% yield; *Z/E* = 88:12; ^1^H-NMR: δ = 7.92 (s, 1H), 7.89 (d, *J* = 8.0 Hz, 2H), 7.62–7.58 (m, 3H), 7.54–7.50 (m, 2H), 6.93 (d, *J* = 8.0 Hz, 2H), 4.52 (s, 2H), 3.85 (s, 3H), 3.51 (s, 3H) ppm; ^13^C-NMR: δ = 167.3, 161.3, 146.5, 139.6, 133.8, 131.7, 129.1, 128.7, 126.3, 118.1, 114.4, 55.6, 55.5, 52.3 ppm; ESI-HRMS: calcd. For C_18_H_18_O_5_S+Na 369.0773, found 369.0771.

*(Z)-Methyl 3-(3,4-dimethoxyphenyl)-2-[(phenylsulfonyl)methyl]acrylate* (**3g**). Viscous liquid; 98% yield; *Z/E* = 90:10; ^1^H-NMR: δ = 7.93 (s, 1H), 7.90 (d, *J* = 8.0 Hz, 2H), 7.65–7.62 (m, 1H), 7.55–7.51 (m, 2H), 7.43 (s, 1H), 7.20–7.17 (m, 1H), 6.90 (d, *J* = 8.0 Hz, 1H), 4.53 (s, 2H), 3.97 (s, 3H), 3.93 (s, 3H), 3.50 (s, 3H), ppm; ^13^C-NMR: δ = 166.1, 149.8, 148.1, 145.8, 138.6, 132.7, 128.0, 127.6, 125.5, 122.7, 117.2, 111.5, 110.1, 55.3, 55.0, 54.8, 51.2 ppm; ESI-HRMS: calcd. For C_19_H_20_O_6_S+K 415.0618, found 415.0618.

*(Z)-Methyl 2-[(phenylsulfonyl)methyl]-3-(m-tolyl)acrylate* (**3h**). Colourless liquid; 96% yield; *Z/E* = 88:12; ^1^H-NMR: δ = 7.88 (s, 1H), 7.81 (d, *J* = 8.0 Hz, 2H), 7.59–7.54 (m, 1H), 7.47–7.43 (m, 2H), 7.24–7.20 (m, 2H), 7.16–7.13 (m, 2H), 4.47 (s, 2H), 3.58 (s, 3H), 2.31(s, 3H) ppm; ^13^C-NMR: δ = 165.9, 145.5, 138.3, 137.4, 132.6, 129.5, 128.8, 127.7, 127.5, 125.1, 119.7, 54.1, 51.4, 20.3 ppm; ESI-HRMS: calcd. For C_18_H_18_O_4_S+Na 353.0823, found 353.0827.

*(Z)-Methyl 2-[(phenylsulfonyl)methyl]-3-(p-tolyl)acrylate* (**3i**). Colourless liquid; 92% yield; *Z/E* = 96:4; ^1^H-NMR: δ = 7.93 (s, 1H), 7.87 (d, *J* = 8.0 Hz, 2H), 7.63–7.60 (m, 1H), 7.52–7.48 (m, 2H), 7.43 (d, *J* = 8.0 Hz, 2H), 7.19 (d, *J* = 8.0 Hz, 2H), 4.50 (s, 2H), 3.56 (s, 3H), 2.38 (s, 3H) ppm; ^13^C-NMR: δ = 166.0, 145.6, 139.3, 138.5, 132.7, 129.8, 128.5, 128.5, 128.0, 127.6, 118.7, 54.3, 51.3, 20.4 ppm; ESI-HRMS: calcd. For C_18_H_18_O_4_S+Na 353.0823, found 353.0820.

*(Z)-Methyl 3-(naphthalen-2-yl)-2-[(phenylsulfonyl)methyl]acrylate* (**3j**). Colourless liquid; 71% yield; *Z/E* = 96:4; ^1^H-NMR: δ = 8.35 (s, 1H), 7.75 (d, *J* = 8.0 Hz, 2H), 7.62–7.59 (m, 3H), 7.46–7.33 (m, 4H), 7.29–7.26 (m, 1H), 7.21–7.17 (m, 2H), 4.38 (s, 2H), 3.66 (s, 3H) ppm; ^13^C-NMR: δ = 166.7, 144.6, 139.2, 133.4, 133.3, 131.0, 130.7, 129.8, 128.9, 128.6, 128.1, 126.7, 126.4, 126.4, 125.3, 124.3, 123.5, 55.1, 52.6 ppm; ESI-HRMS: calcd. For C_21_H_18_O_4_S+Na 389.0823, found 389.0825.

*(Z)-Methyl 3-(furan-2-yl)-2-[(phenylsulfonyl)methyl]acrylate* (**3k**). Semi-solid; 85% yield; *Z/E* > 99:1; ^1^H-NMR: δ = 7.84 (s, 1H), 7.83–7.82 (m, 1H), 7.53–7.50 (m, 2H), 7.44–7.39 (m, 3H), 6.67 (d, *J* = 4.0 Hz, 1H), 6.40 (m, 1H), 4.79 (s, 2H), 3.59 (s, 3H) ppm;^13^C-NMR: δ = 166.9, 150.0, 145.8, 139.4, 133.6, 130.7, 128.7, 128.7, 119.1, 115.5, 112.3, 55.5, 52.4 ppm; ESI-HRMS: calcd. For C_15_H_14_O_5_S+H 307.0640, found 307.0649.

*(Z)-Methyl 2-[(phenylsulfonyl)methyl]-3-(thiophen-2-yl)acrylate* (**3l**). Light brown liquid; 96% yield; *Z/E* = 81:19; ^1^H-NMR: δ = 8.03 (s, 1H), 7.88 (d, *J* = 8.0 Hz, 1H), 7.60–7.56 (m, 1H), 7.53–7.46 (m, 5H), 7.08–7.06 (m, 1H), 4.61 (s, 2H), 3.50 (s, 3H) ppm; ^13^C-NMR: δ = 165.7, 138.4, 137.3, 135.7, 133.2, 132.8, 129.9, 128.0, 127.6, 126.8, 115.1, 55.0, 51.3 ppm; ESI-HRMS: calcd. For C_15_H_14_O_4_S_2_+H 323.0412, found 323.0417.

*(Z)-Methyl 2-[(phenylsulfonyl)methyl]hex-2-enoate* (**3m**). viscous liquid; 91% yield; *Z/E* = 82:18; ^1^H-NMR: δ = 7.86–7.81 (m, 2H), 7.64–7.61 (m, 1H), 7.54–7.51 (m, 2H), 7.12 (t, *J* = 8.0 Hz, 1H), 4.24 (s, 2H), 3.48 (s, 3H), 2.20–2.14 (m, 2H), 1.49–1.40 (m, 2H), 0.91 (t, *J* = 8.0 Hz, 3H) ppm; ^13^C-NMR: δ = 166.2, 151.9, 139.0, 133.9, 129.2, 128.9, 120.8, 54.2, 52.1, 31.6, 21.7, 14.0 ppm; ESI-HRMS: calcd. For C_14_H_18_O_4_S+Na 305.0823, found 305.0829.

*(E)-3-Phenyl-2-[(phenylsulfonyl)methyl]acrylonitrile* (**3n**). Colourless liquid; 99% yield; *Z/E* < 1:99; ^1^H-NMR: δ = 7.94–7.92 (m, 2H), 7.74–7.68 (m, 3H), 7.63–7.59 (m, 2H), 7.47–7.41 (m, 3H), 7.09 (s, 1H), 4.05 (s, 2H) ppm; ^13^C-NMR: δ = 151.9, 137.6, 134.7, 132.5, 131.7, 129.6, 129.3, 129.1, 128.8, 117.1, 98.0, 61.4 ppm; ESI-HRMS: calcd. For C_16_H_13_NO_2_S+Na 306.0565, found 306.0566.

*(Z)-Methyl 3-phenyl-2-(tosylmethyl)acrylate* (**3o**). Colourless liquid; 95% yield; *Z/E* = 84:16; ^1^H-NMR: δ = 7.93 (s, 1H), 7.71 (d, *J* = 8.0 Hz, 2H), 7.47 (m, 2H), 7.37 (m, 3H), 7.27 (d, *J* = 8.0 Hz, 2H), 4.48 (s, 2H), 3.62 (s, 3H), 2.42 (s, 3H) ppm; ^13^C-NMR: δ = 167.1, 146.3, 144.8, 136.3, 133.8, 129.7, 129.3, 128.8, 128.6, 121.2, 55.2, 52.5, 21.7 ppm; ESI-HRMS: calcd. For C_18_H_18_O_4_S+Na 353.0823, found 353.0827.

## 4. Conclusions

In summary, we have established a method for the allylic sulfonylation of MBH carbonates with sodium sulfinates under catalyst-free reaction conditions. A series of functionalized trisubstituted allylic sulfones were rapidly generated in good to excellent yields (71%–99%) with good to high selectivity (*Z/E* from 79:21 to >99:1). Compared with known synthetic methods, the current protocol features mild reaction temperature (40 °C), high efficiency (full conversion within 2 h) and easily available reagents. Thus, it should provide an efficient and facile access to the trisubstituted allylic sulfones. Further studies on expanding the substrate scope and chemical transformations of the trisubstituted allylic sulfones are currently underway.

## Supplementary Materials

Supplementary materials can be accessed at: http://www.mdpi.com/1420-3049/20/05/8213/s1.
