# Supplementary material for: A Facile and Mild Synthesis of Trisubstituted Allylic Sulfones from Morita-Baylis-Hillman Carbonates
Source: Molecules. 2015 May 7;20(5):8213–22. doi: 10.3390/molecules20058213 (PMC6272667; doi:10.3390/molecules20058213)
Supplement: Supplementary file 1 [file molecules-20-08213-s001.pdf]

Supplementary Materials

^{1}H- and ^{13}C-NMR spectra

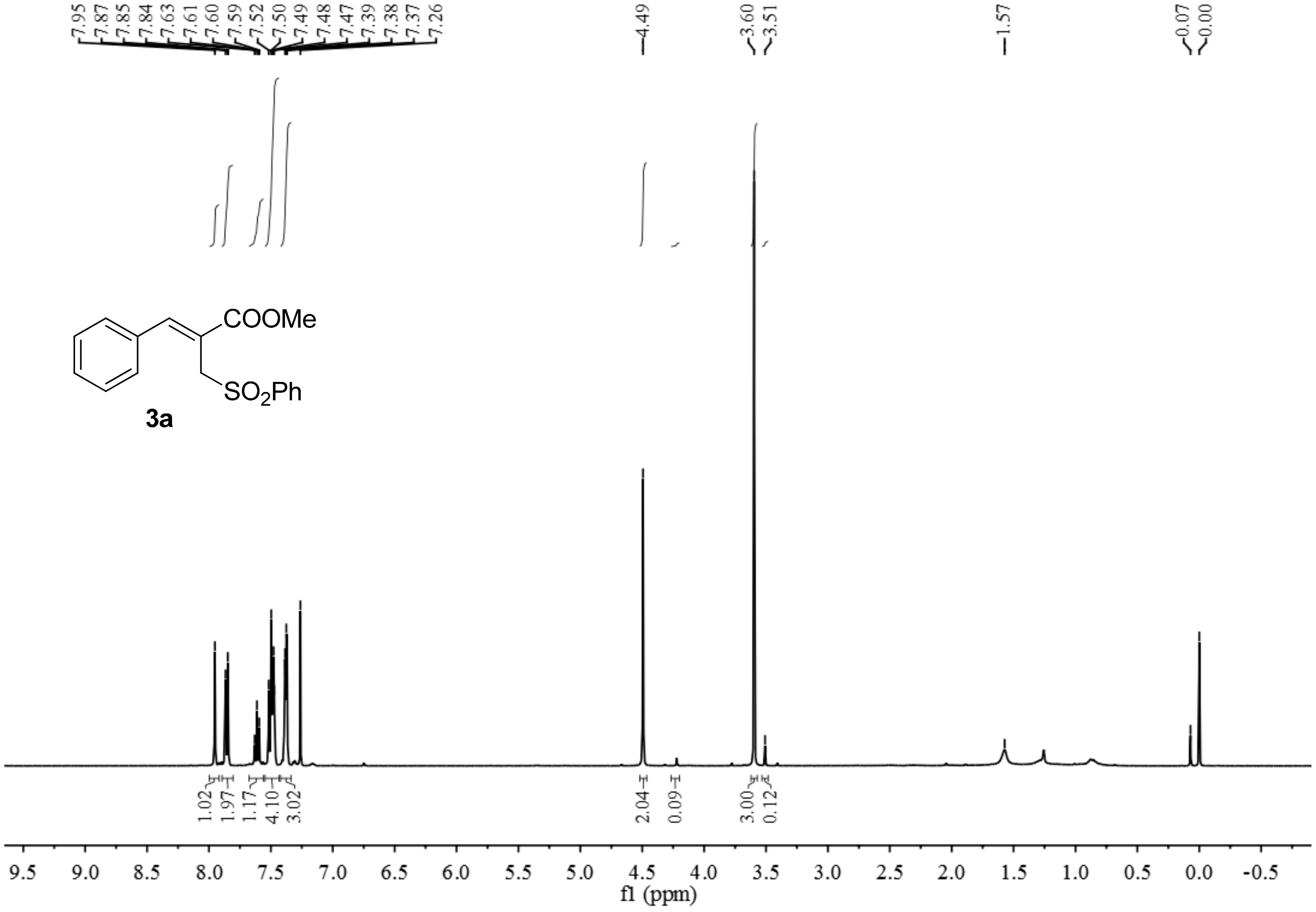

Figure S1. ^{1}H-NMR spectrum of compound **3a**.

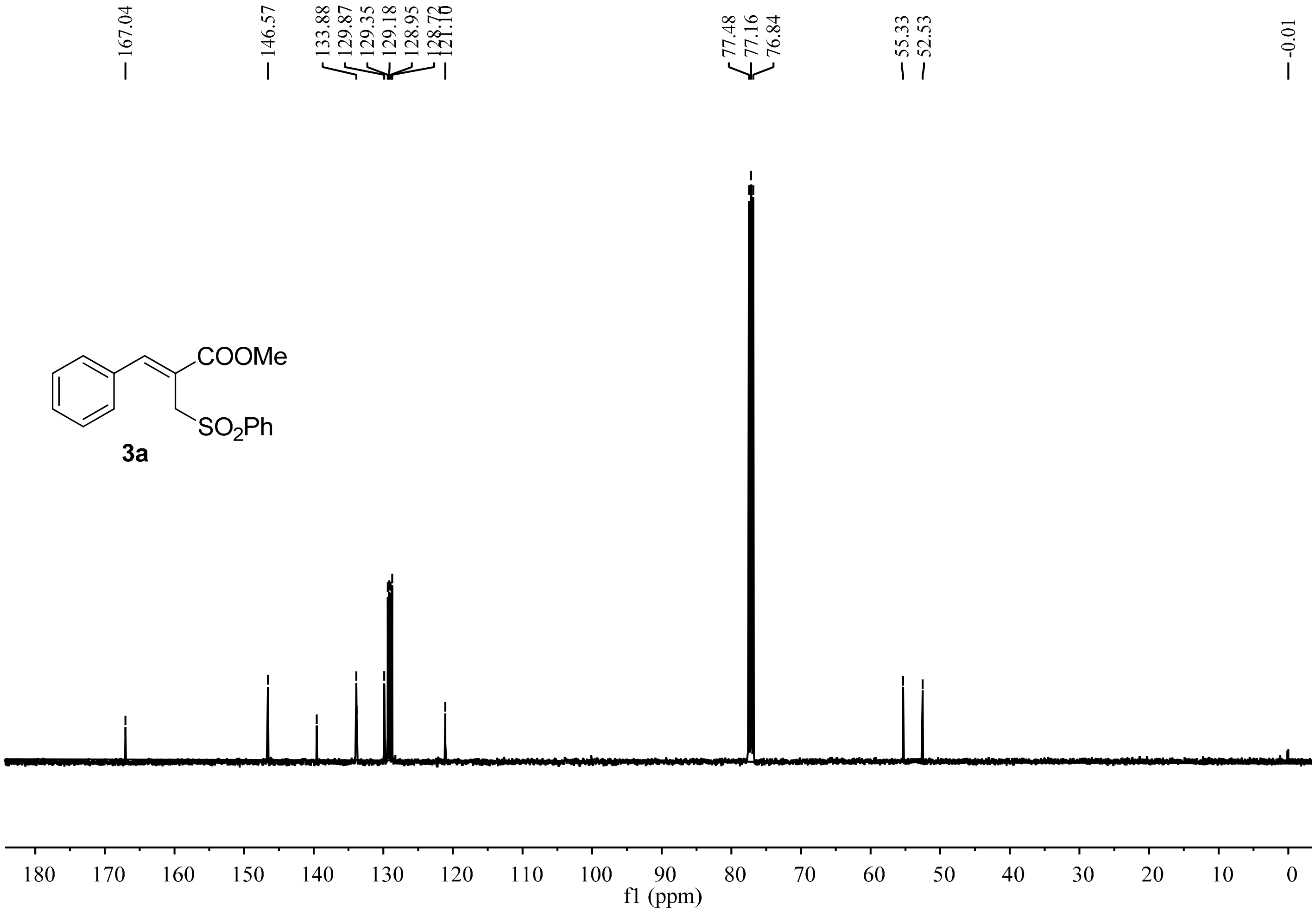

Figure S2. ^{13}C-NMR spectrum of compound **3a**.

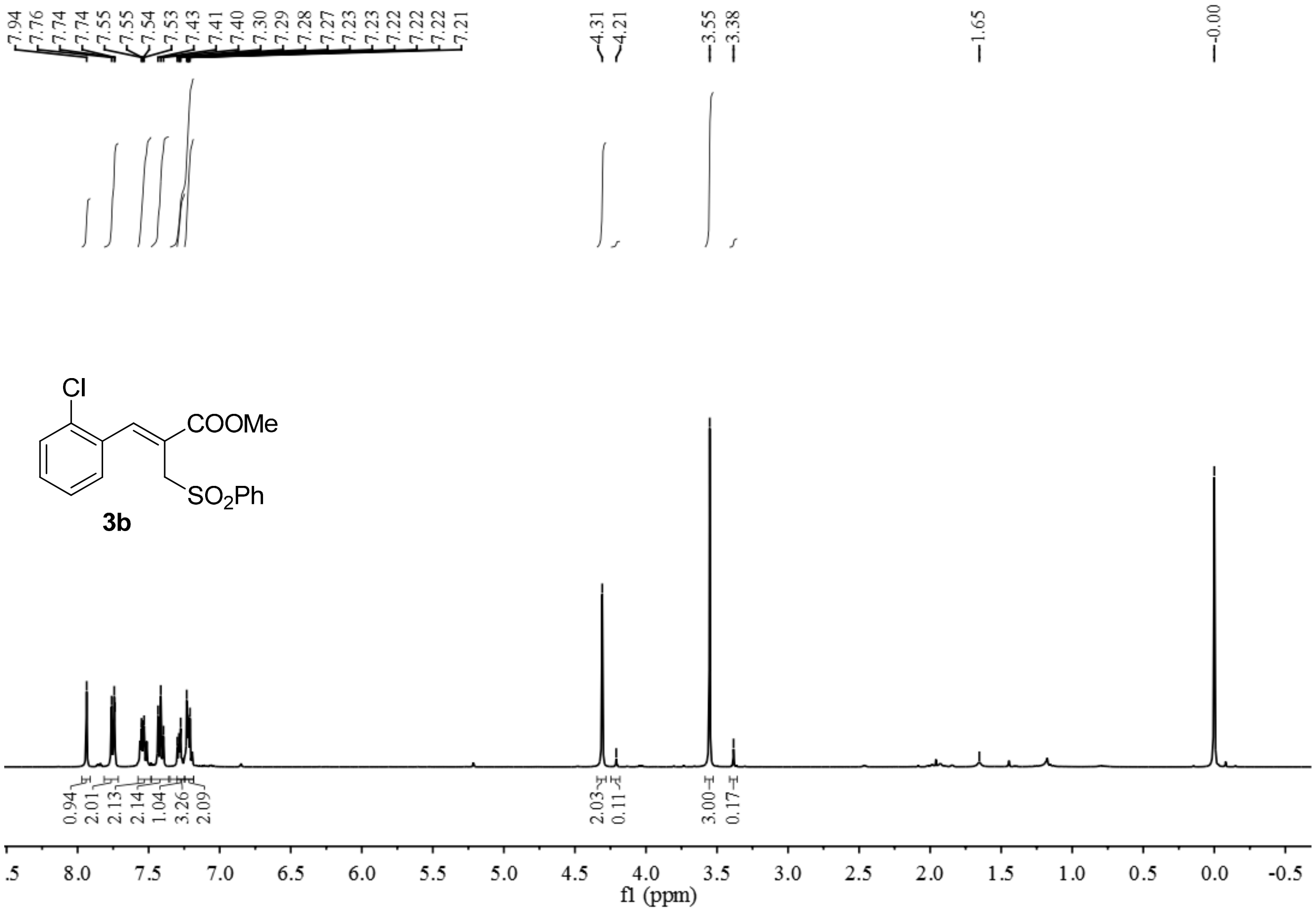

Figure S3. ^{1}H NMR spectrum of compound **3b**.

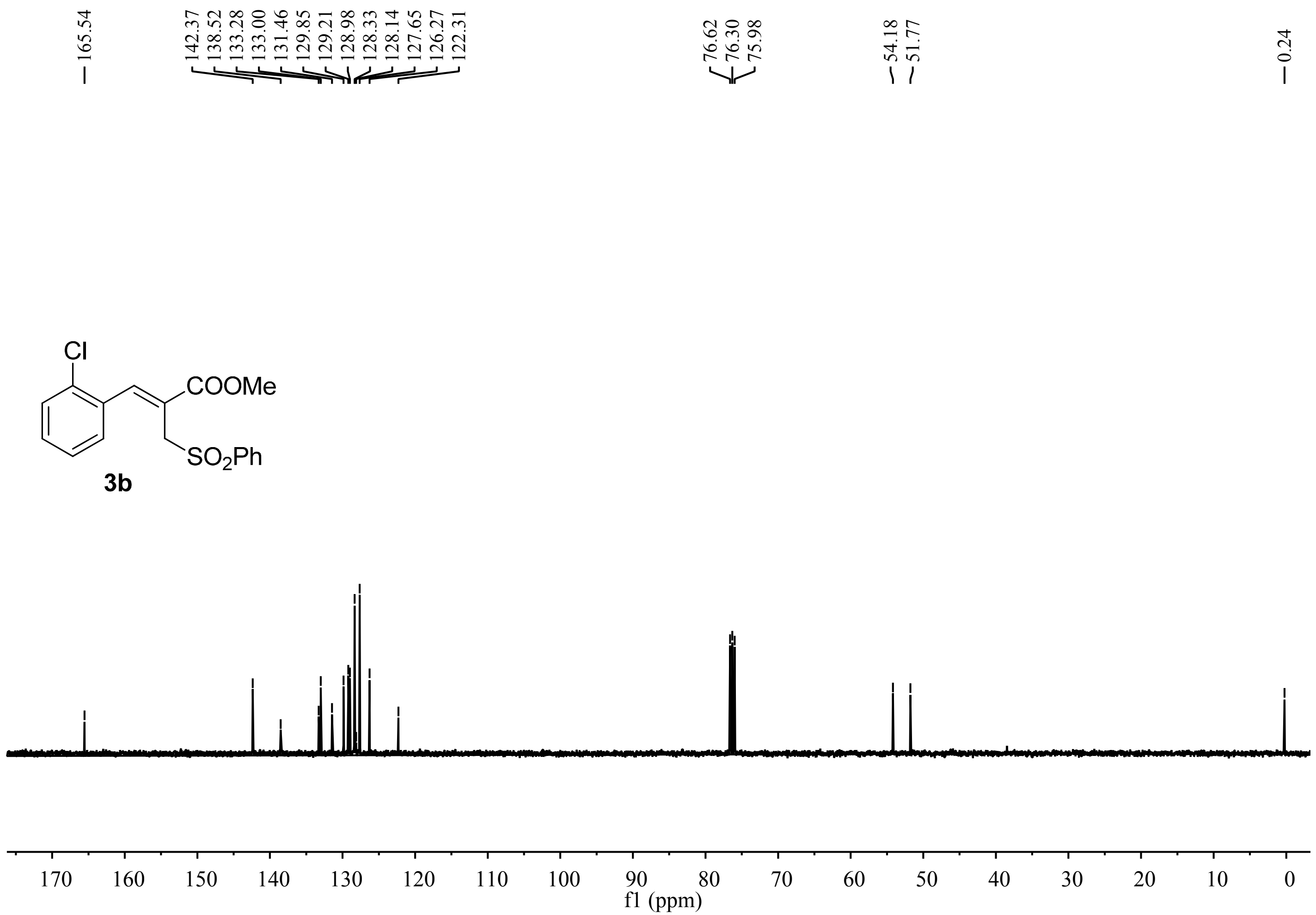

Figure S4. ^{13}C-NMR spectrum of compound **3b**.

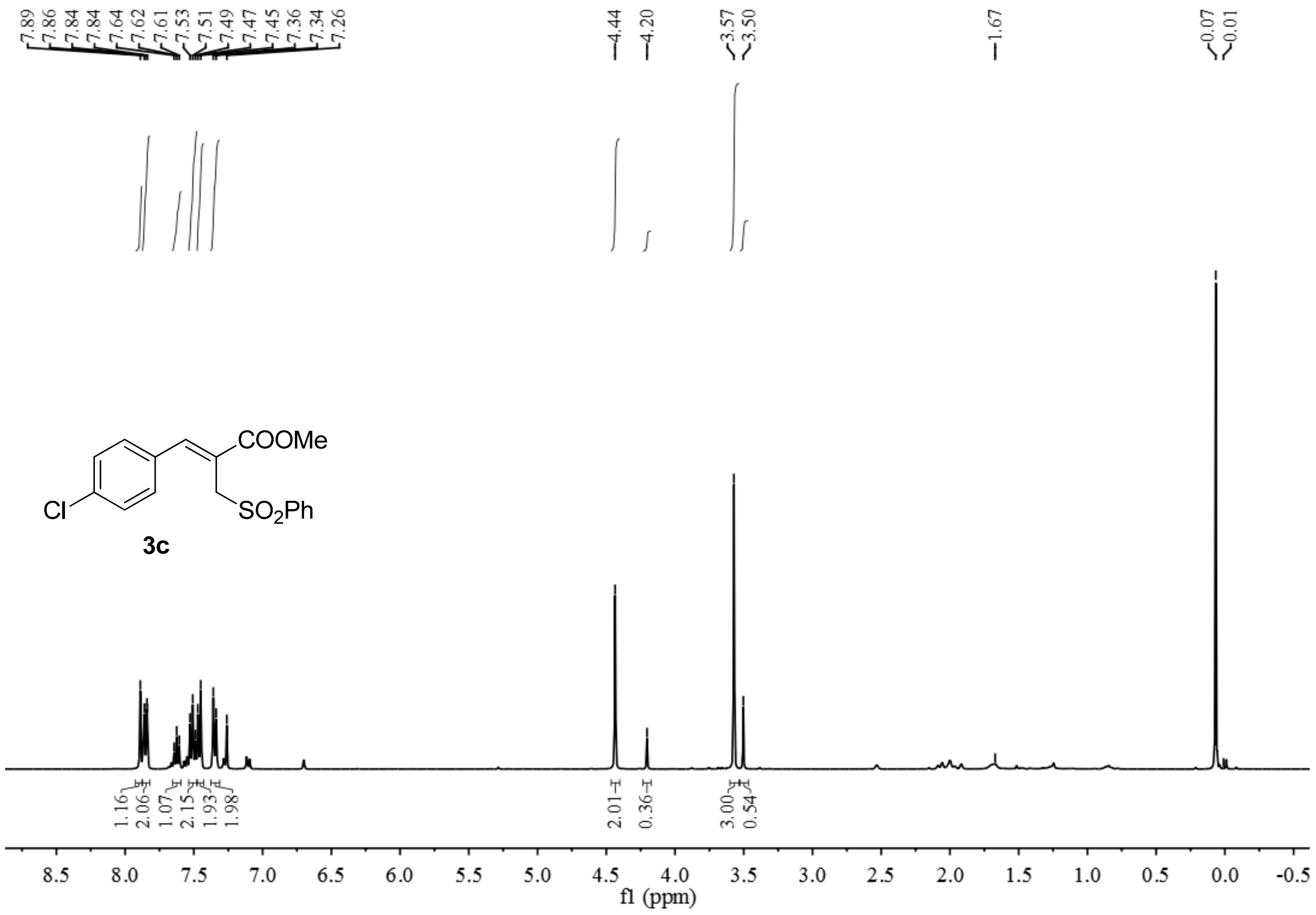

Figure S5. ^{1}H-NMR spectrum of compound **3c**.

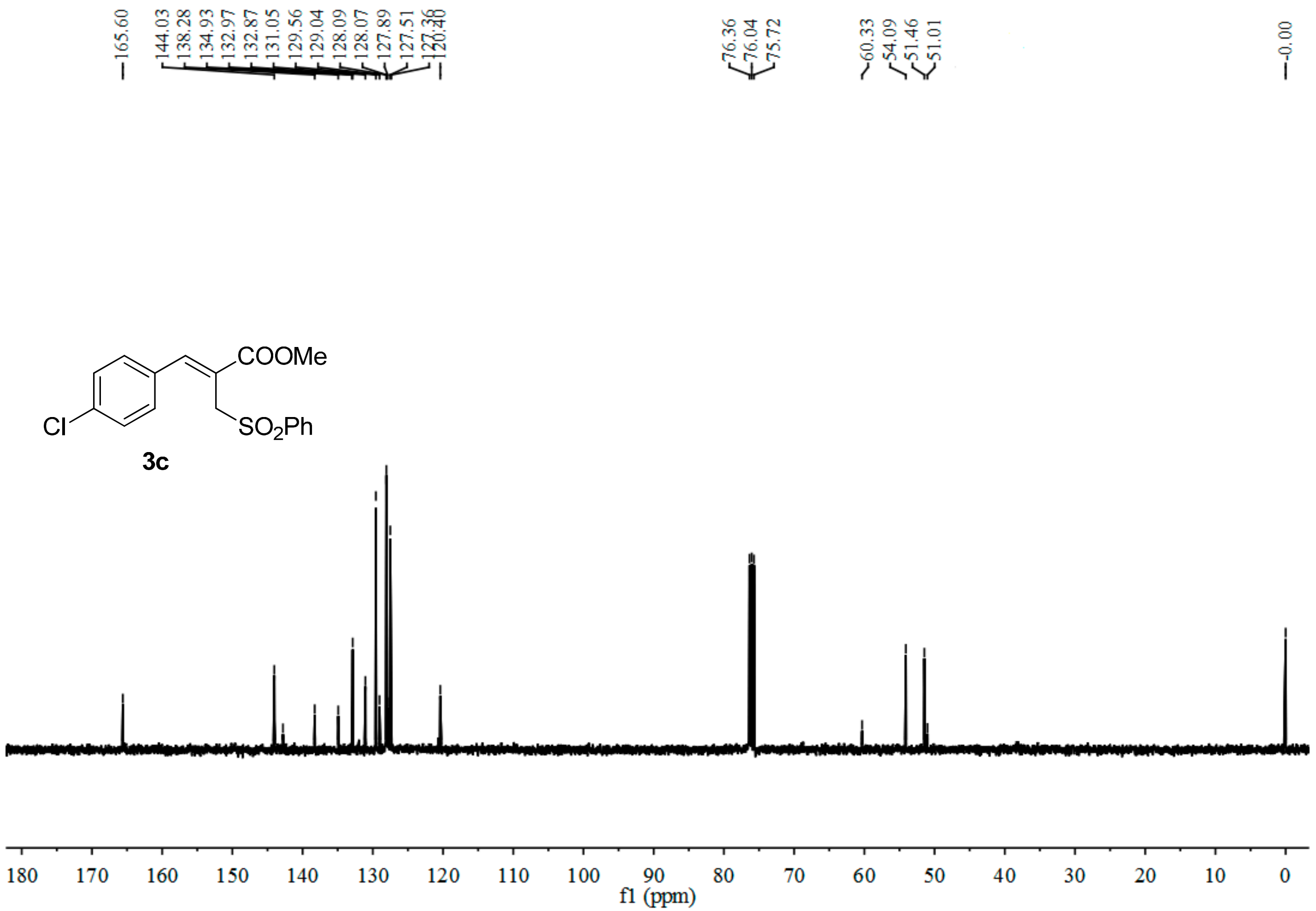

Figure S6. ^{13}C-NMR spectrum of compound **3c**.

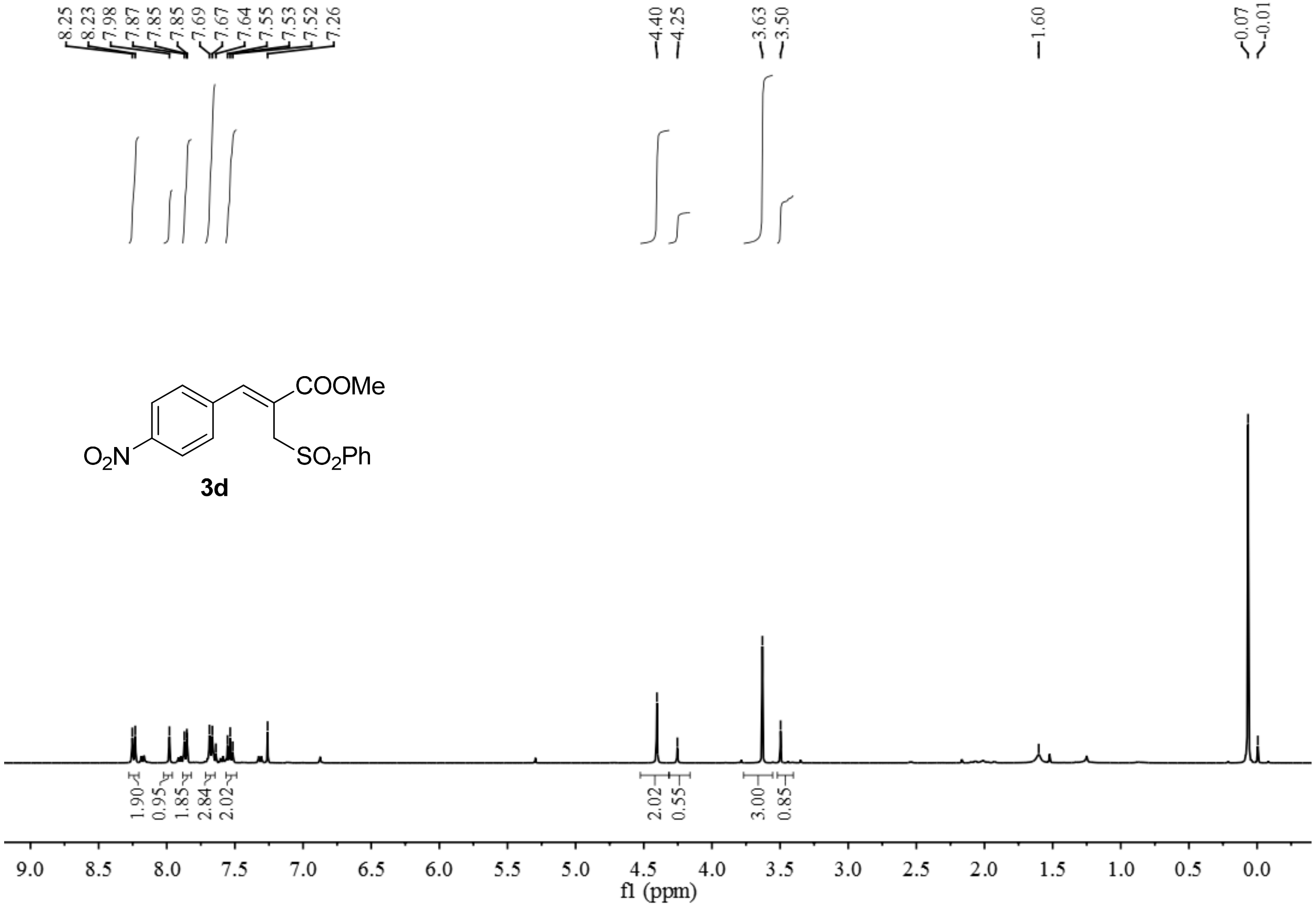

Figure S7. ^{1}H-NMR spectrum of compound **3d**.

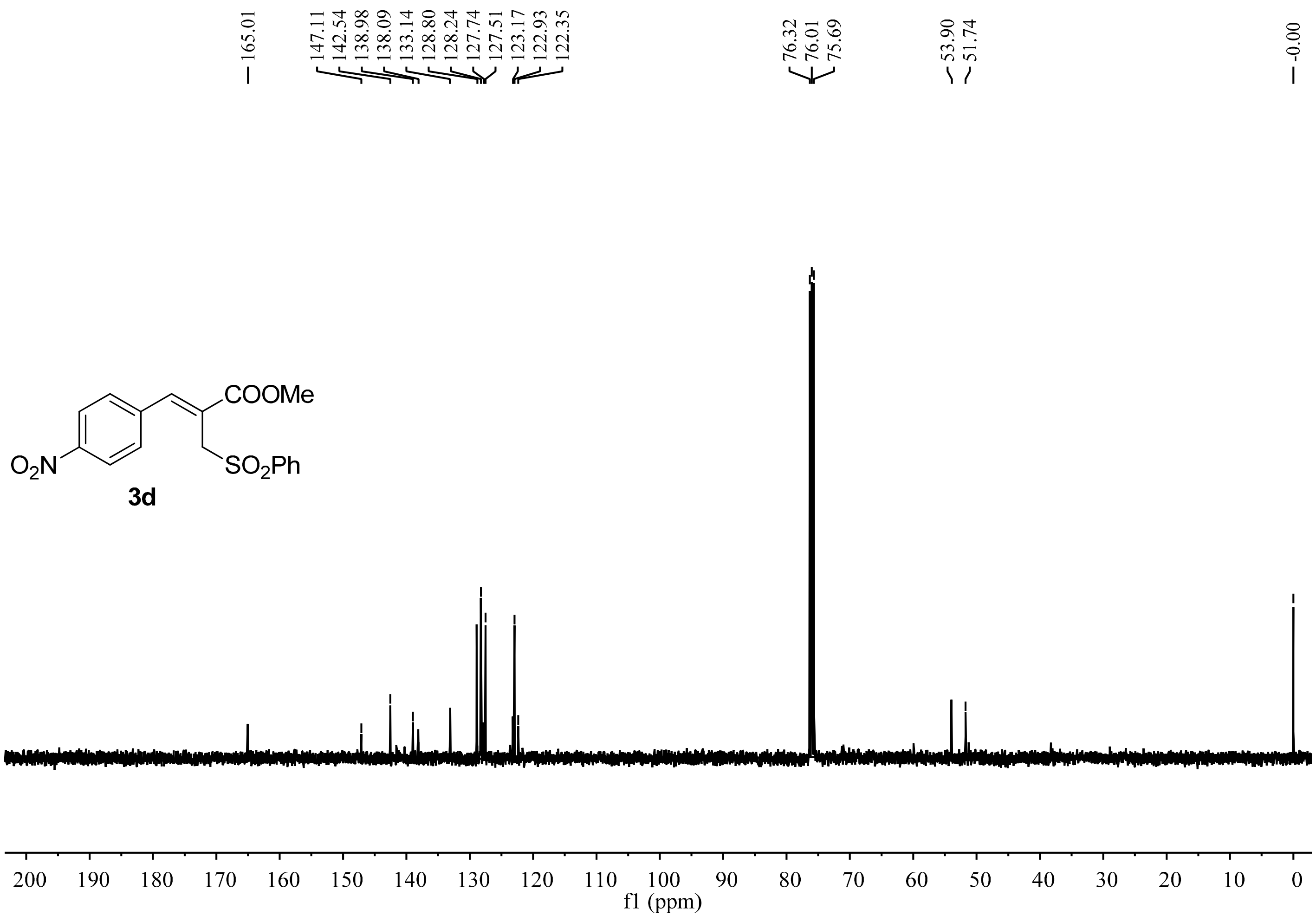

Figure S8. ^{13}C-NMR spectrum of compound **3d**.

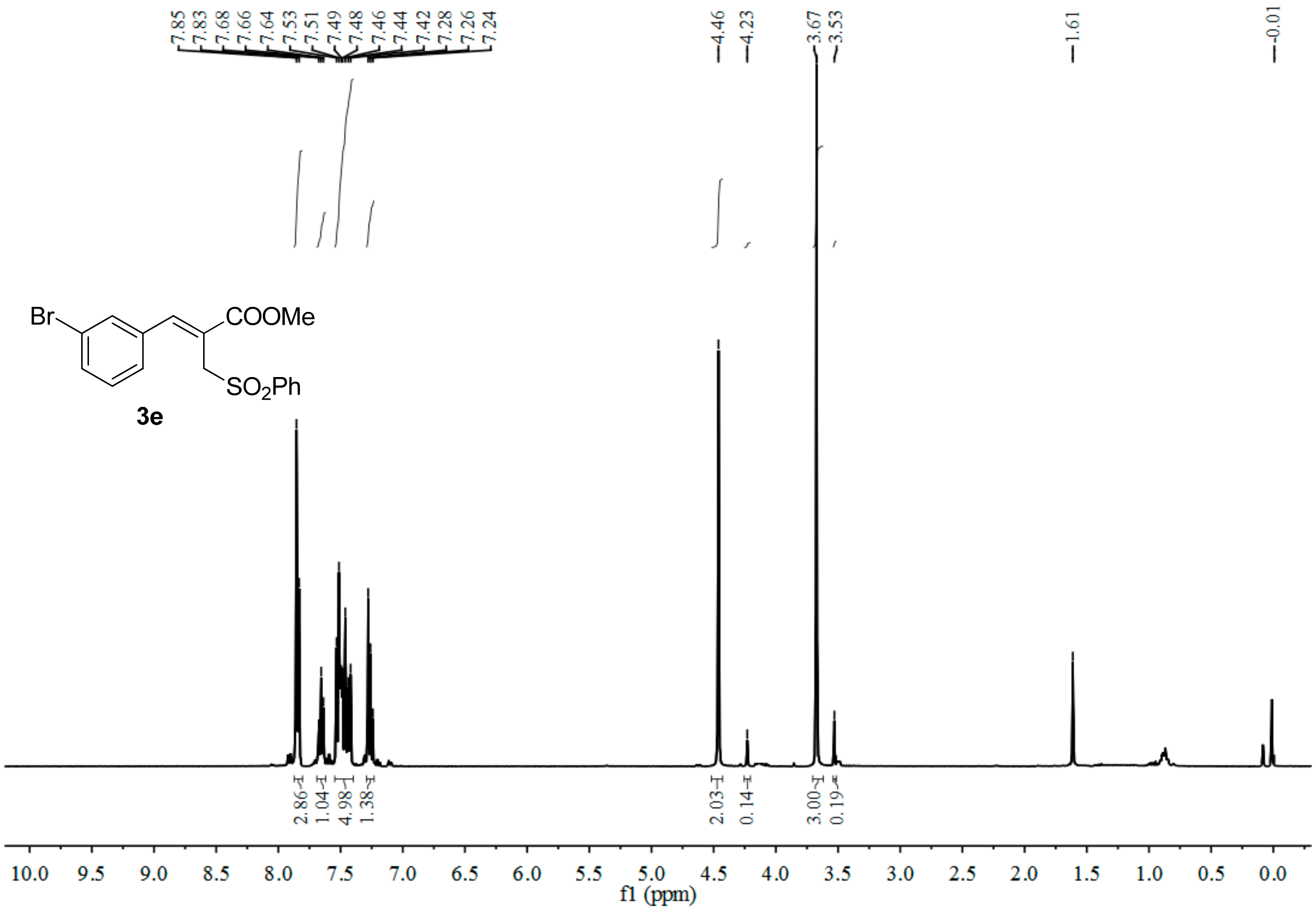

Figure S9. ^{1}H-NMR spectrum of compound **3e**.

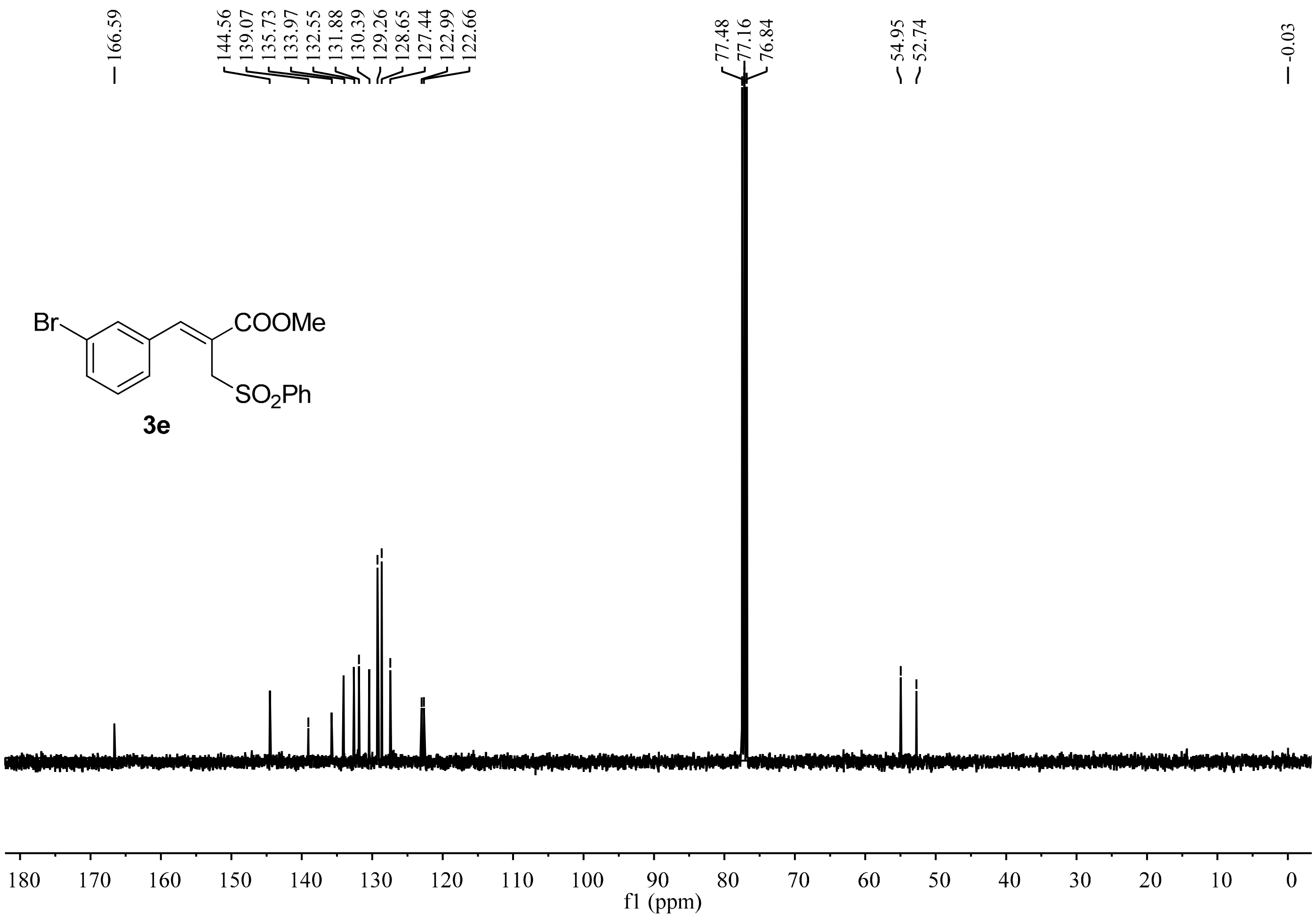

Figure S10. ^{13}C-NMR spectrum of compound **3e**.

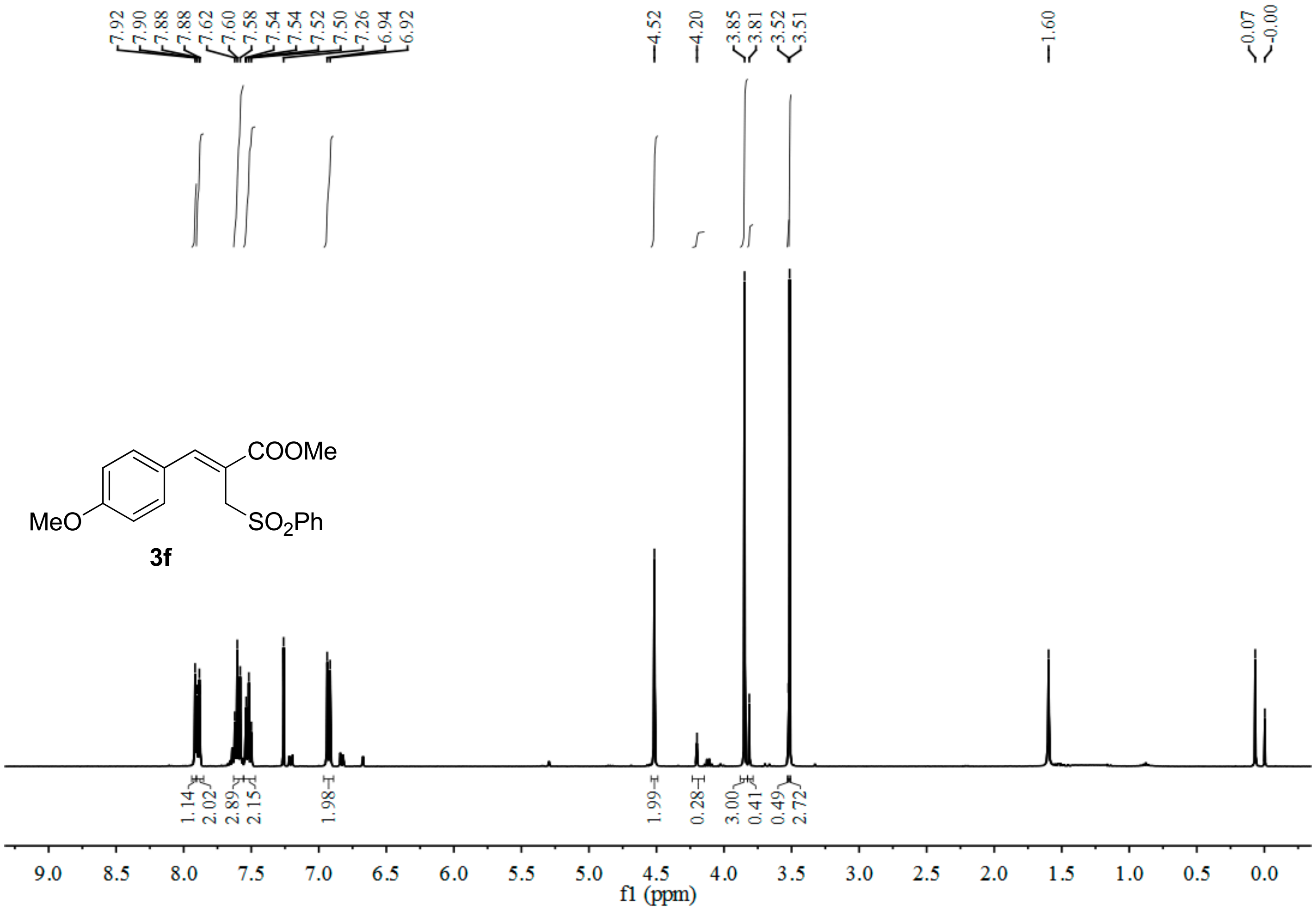

Figure S11. ^{1}H-NMR spectrum of compound **3f**.

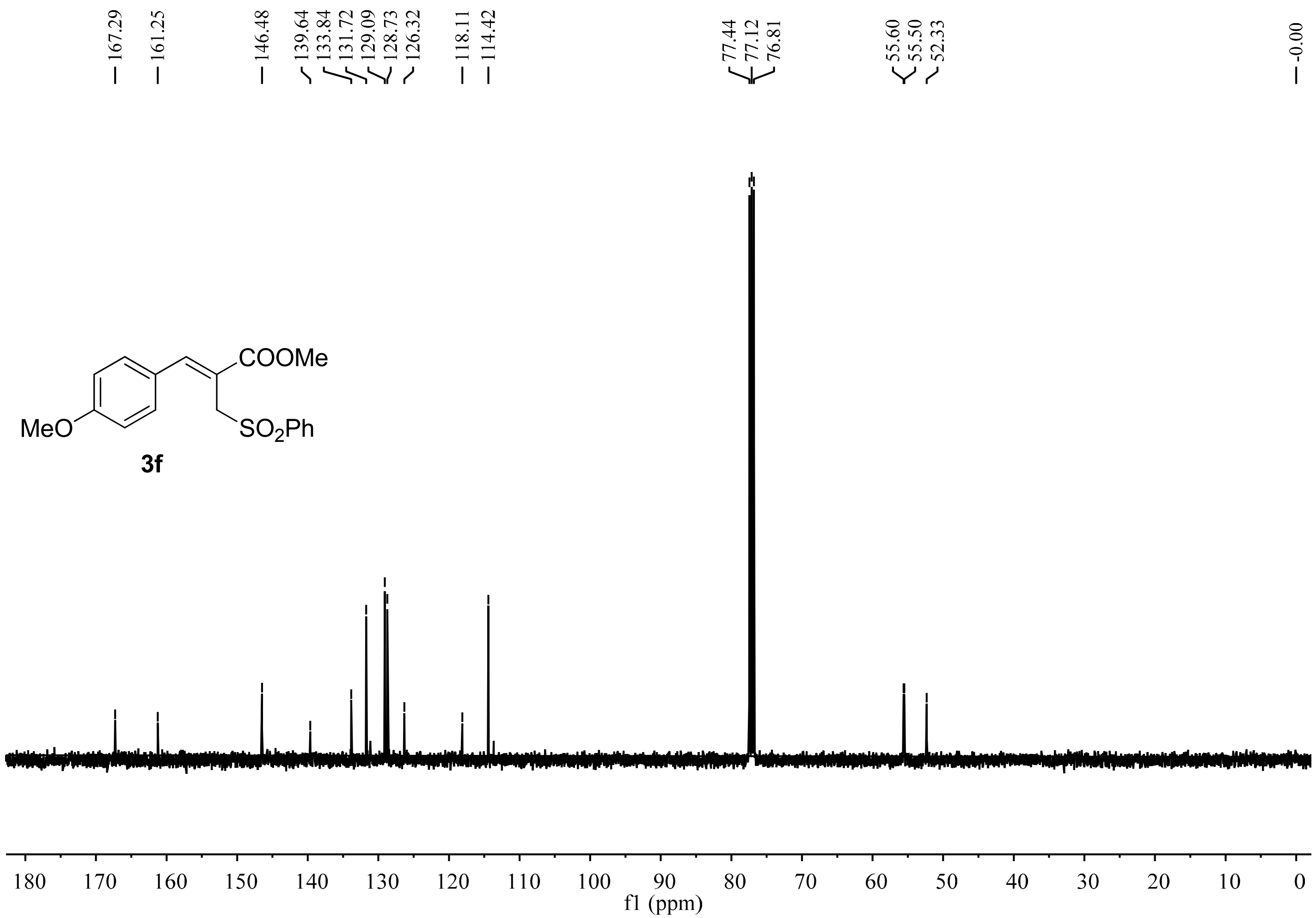

Figure S12. ^{13}C-NMR spectrum of compound **3f**.

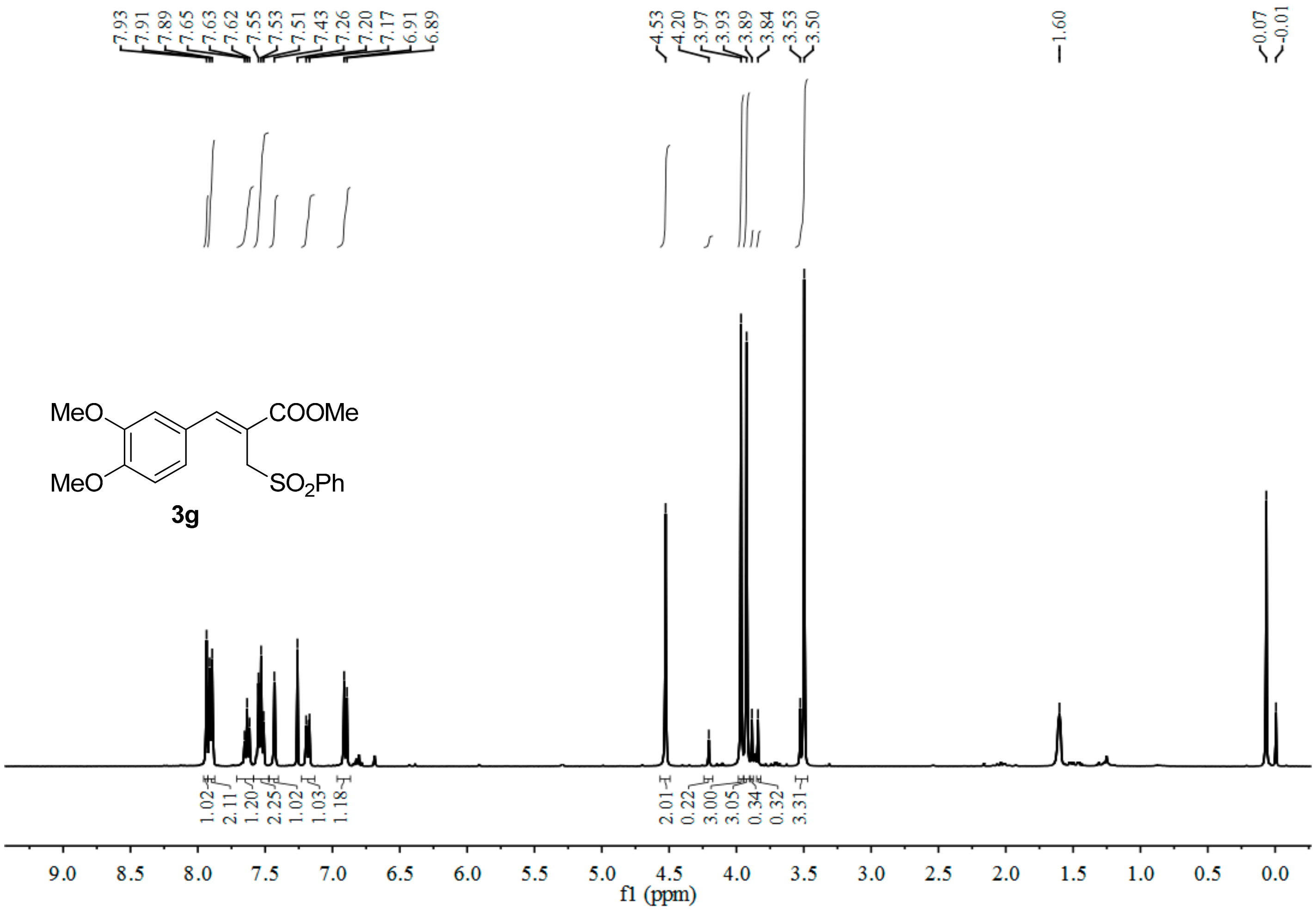

Figure S13. ^{1}H-NMR spectrum of compound **3g**.

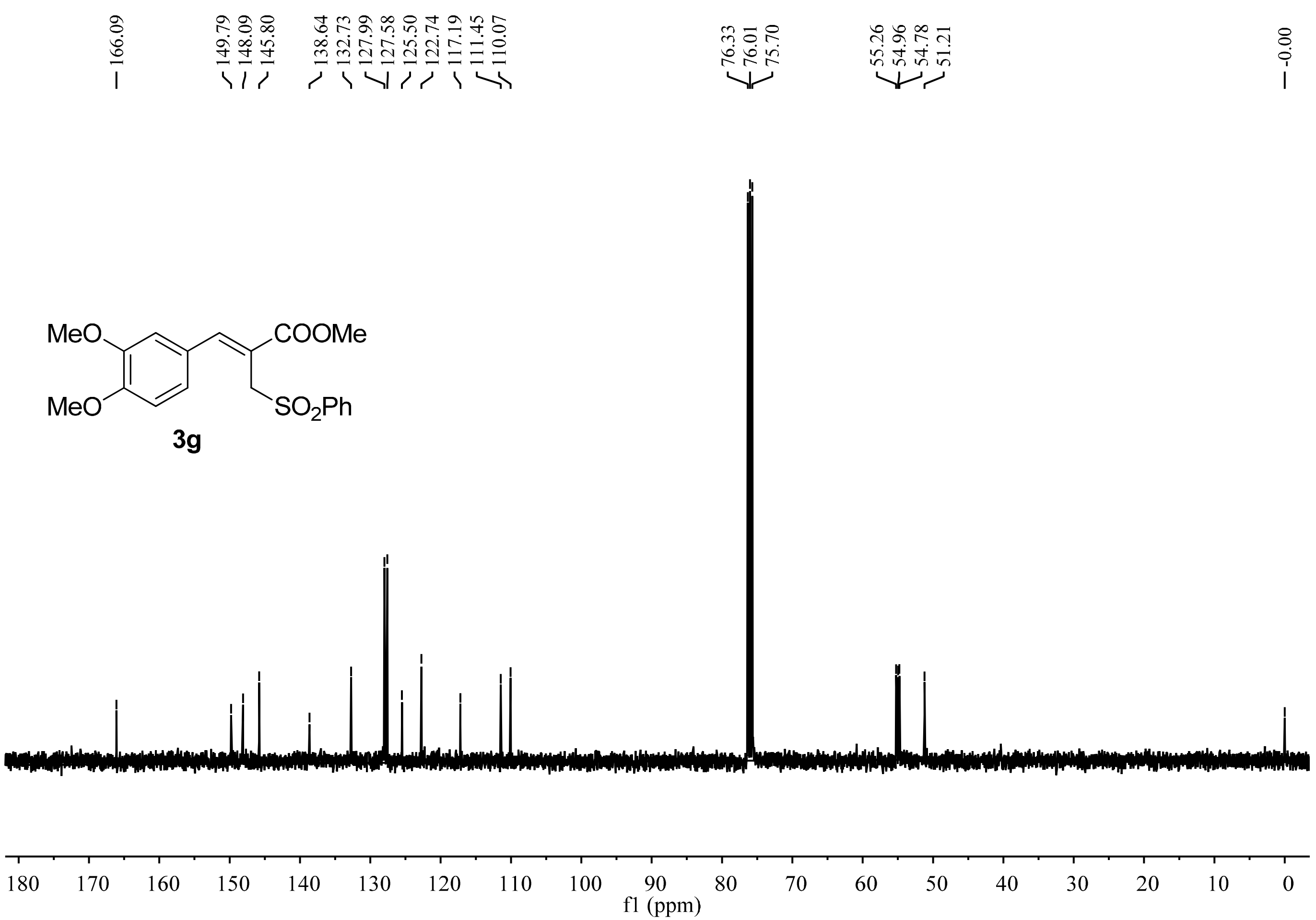

Figure S14. ^{13}C-NMR spectrum of compound **3g**.

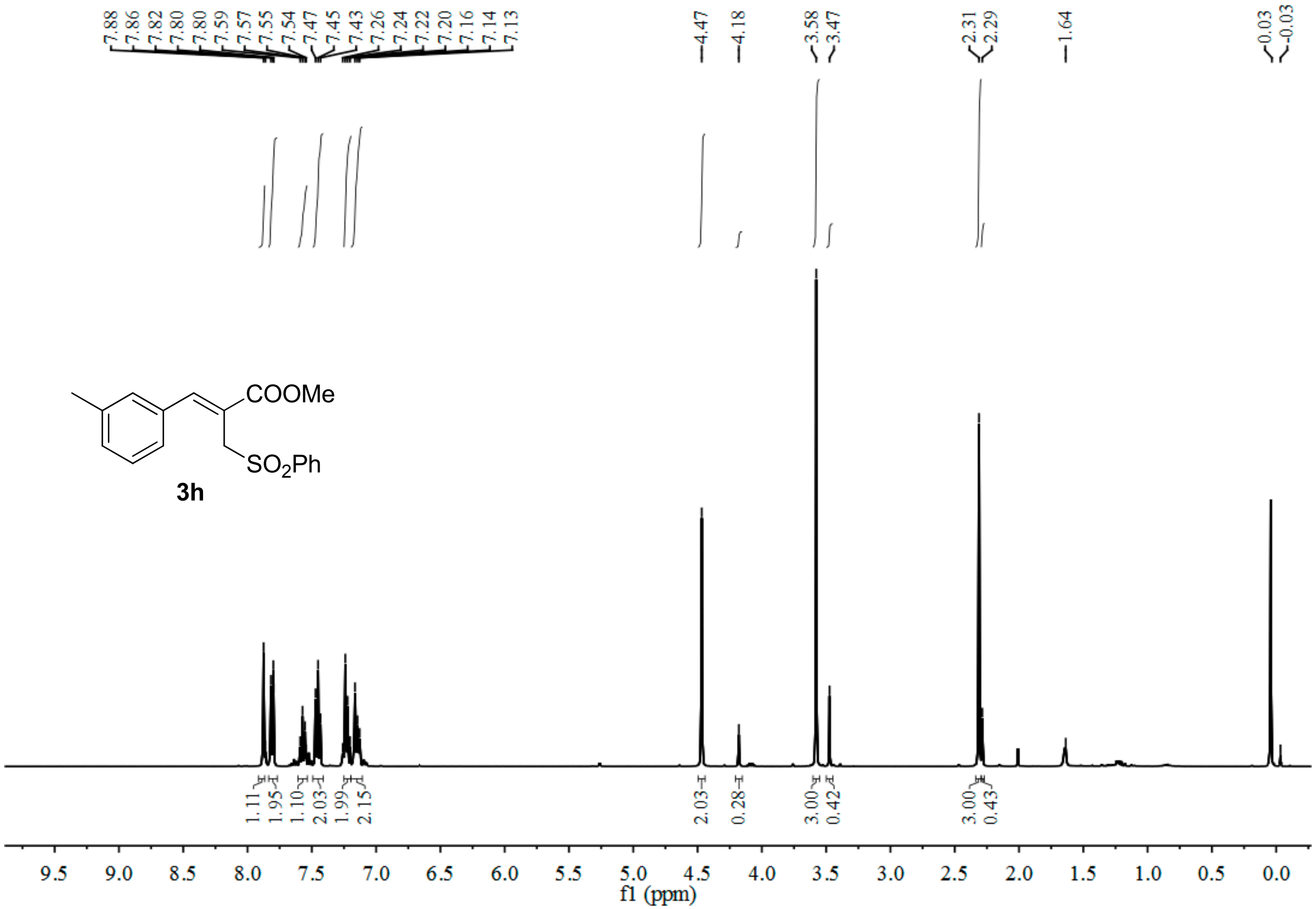

Figure S15. ^{1}H-NMR spectrum of compound **3h**.

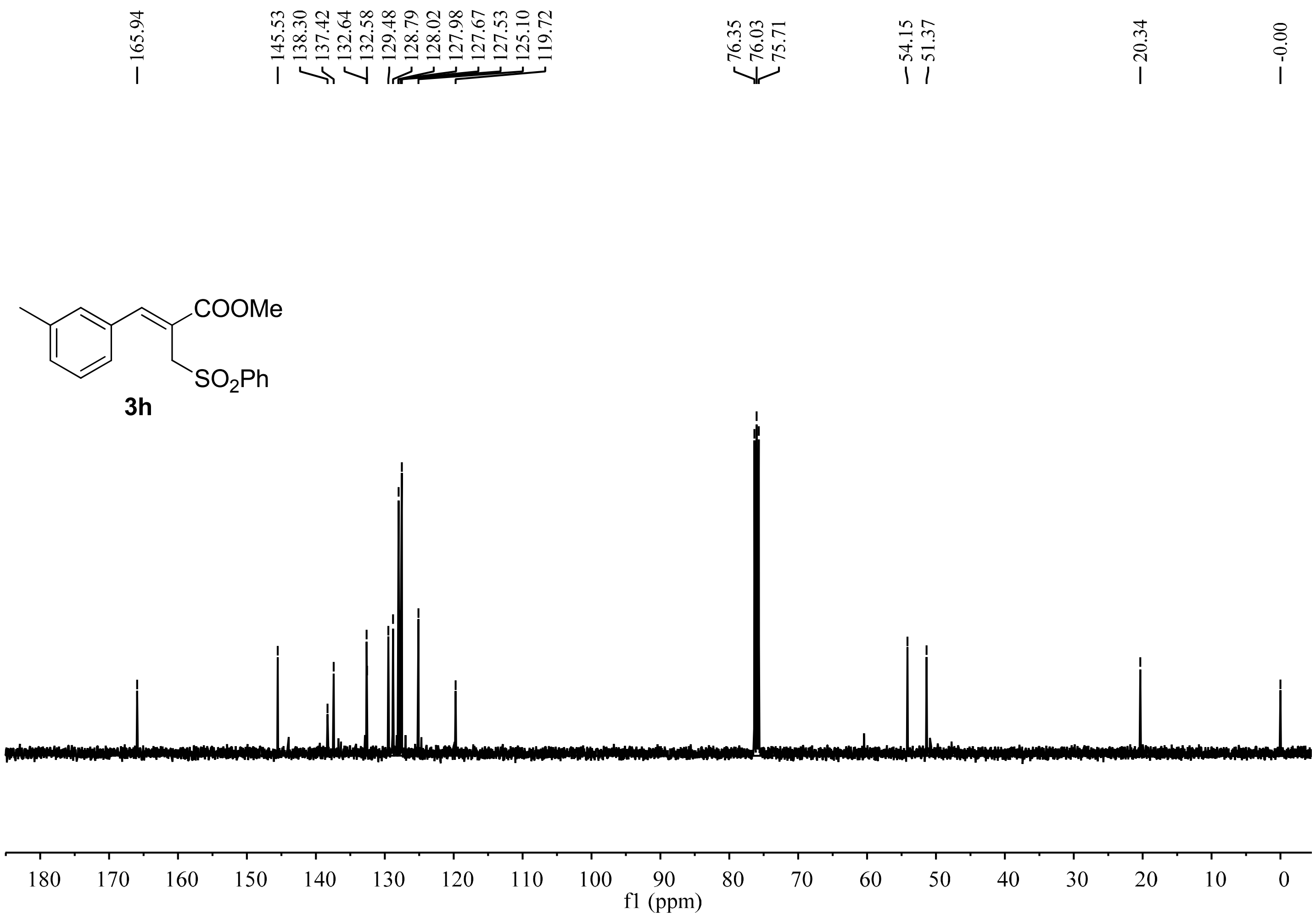

Figure S16. ^{13}C-NMR spectrum of compound **3h**.

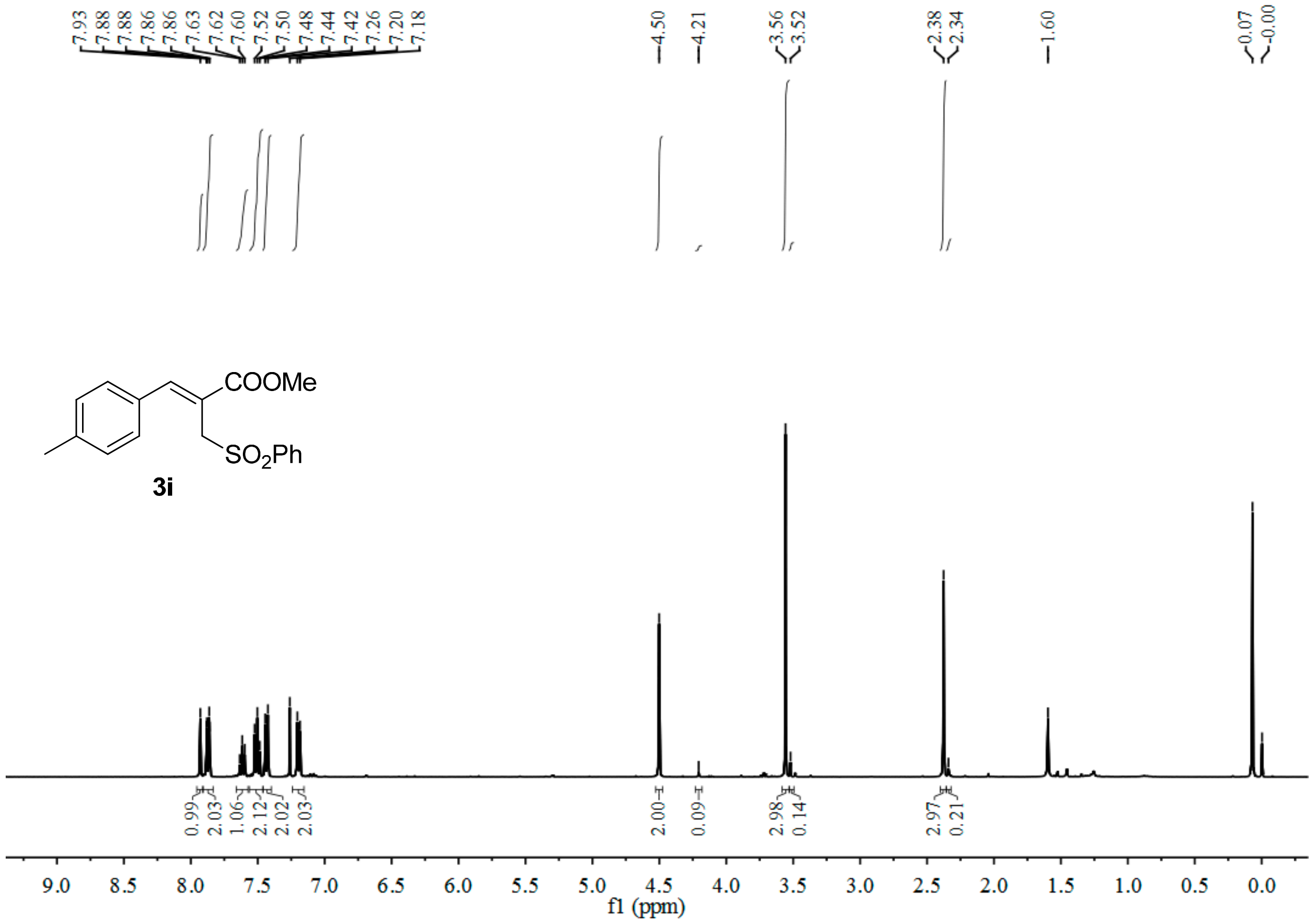

Figure S17. ^{1}H-NMR spectrum of compound **3i**.

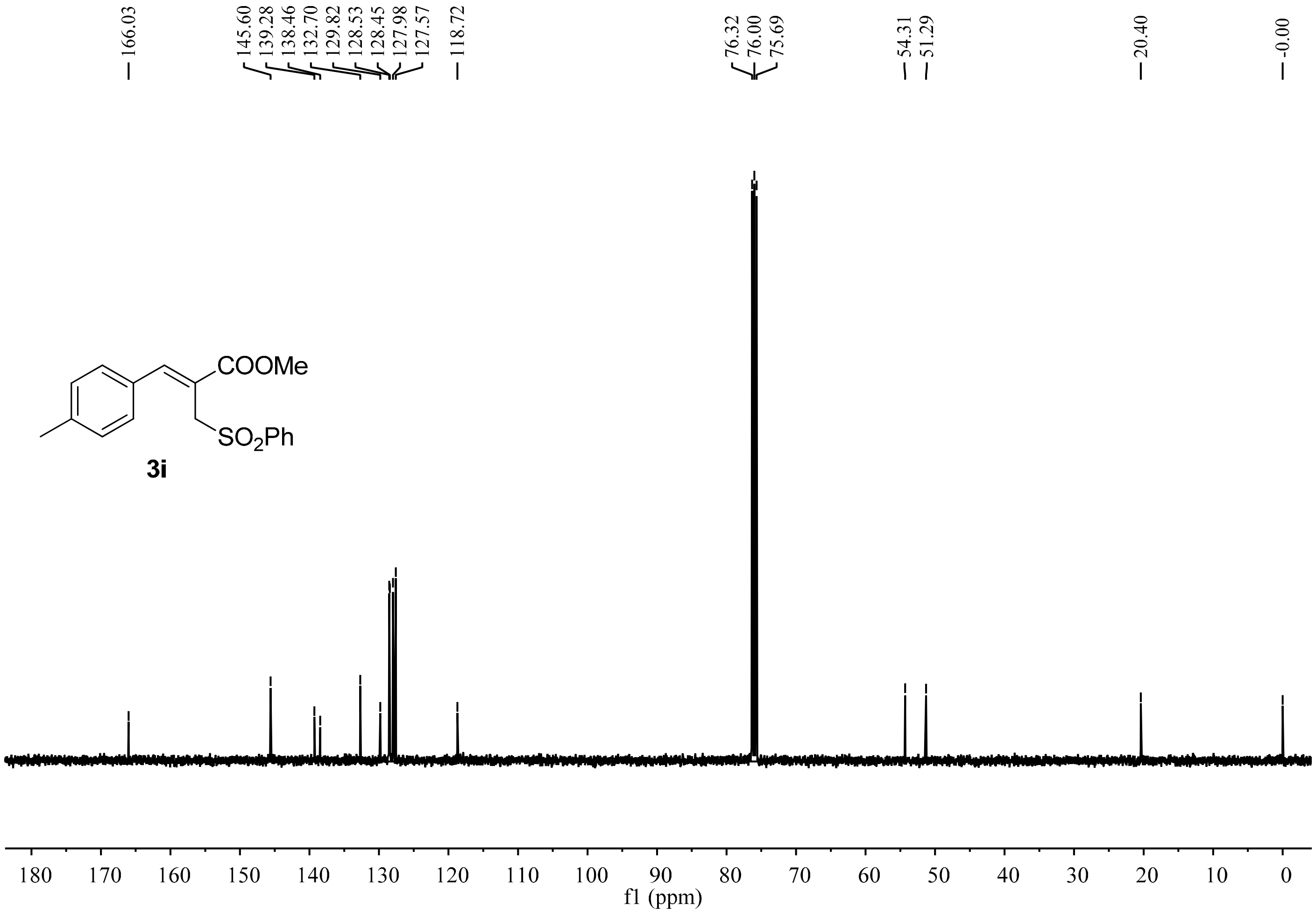

Figure S18. ^{13}C-NMR spectrum of compound **3i**.

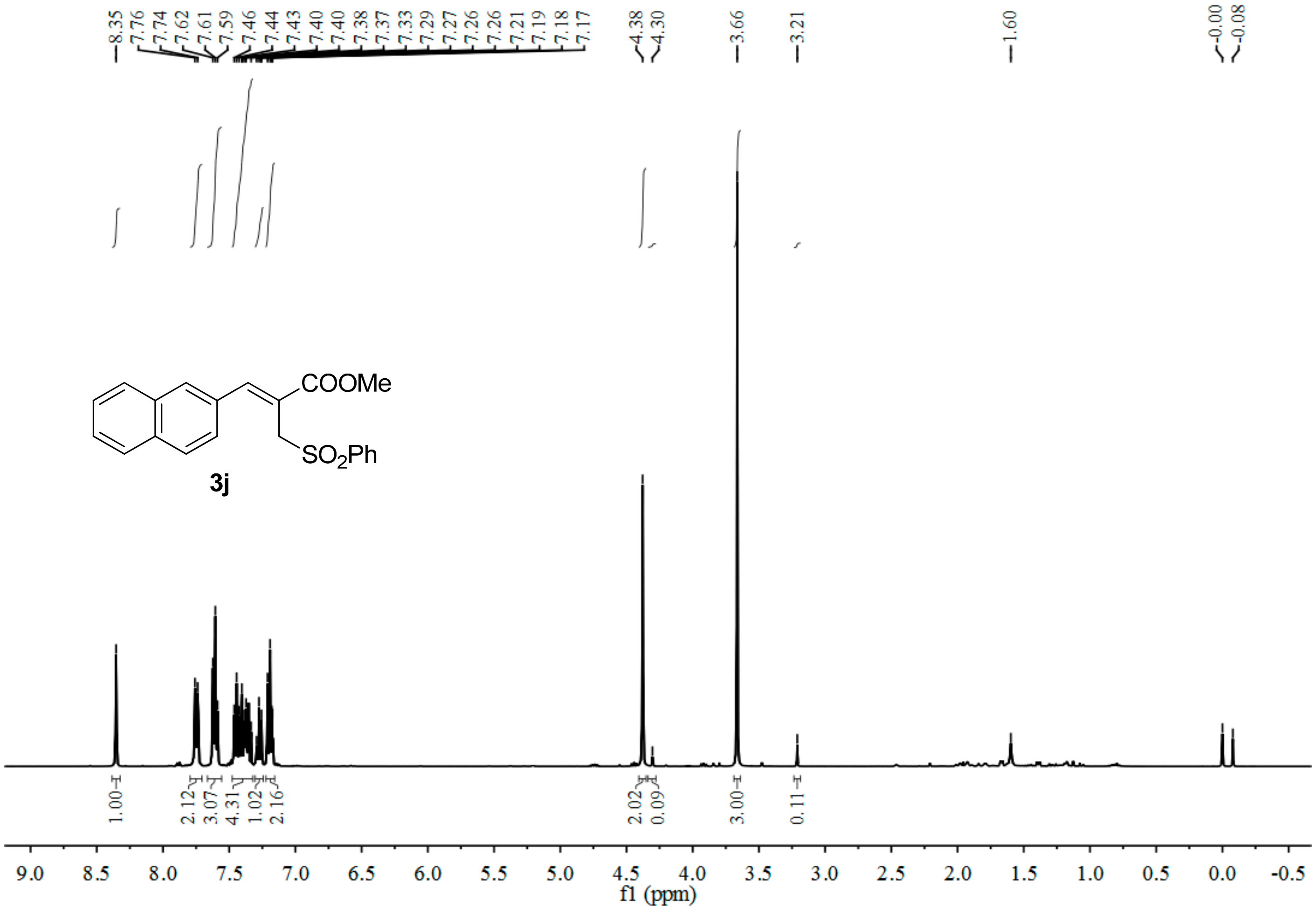

Figure S19. ^{1}H-NMR spectrum of compound **3j**.

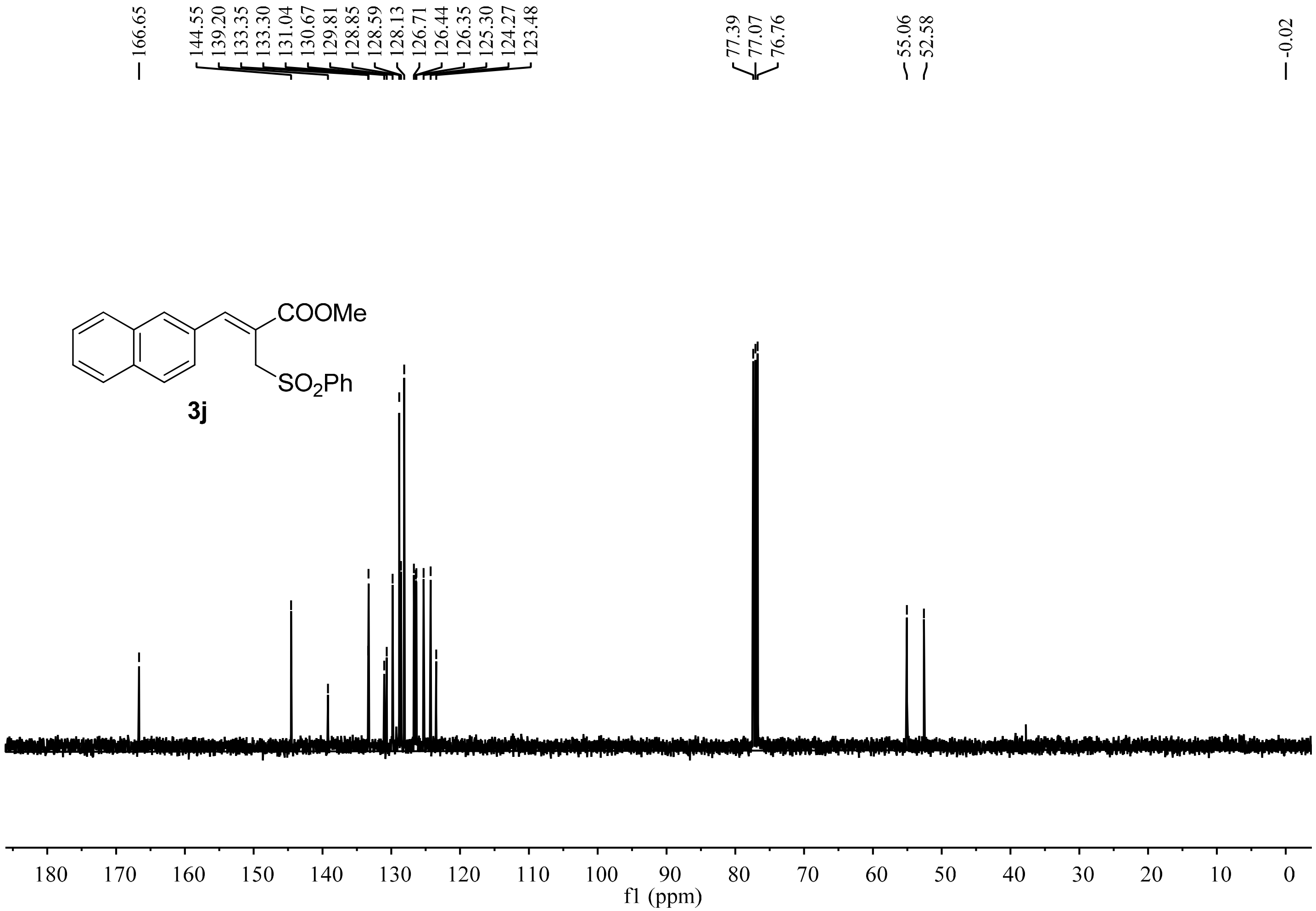

Figure S20. ^{13}C-NMR spectrum of compound **3j**.

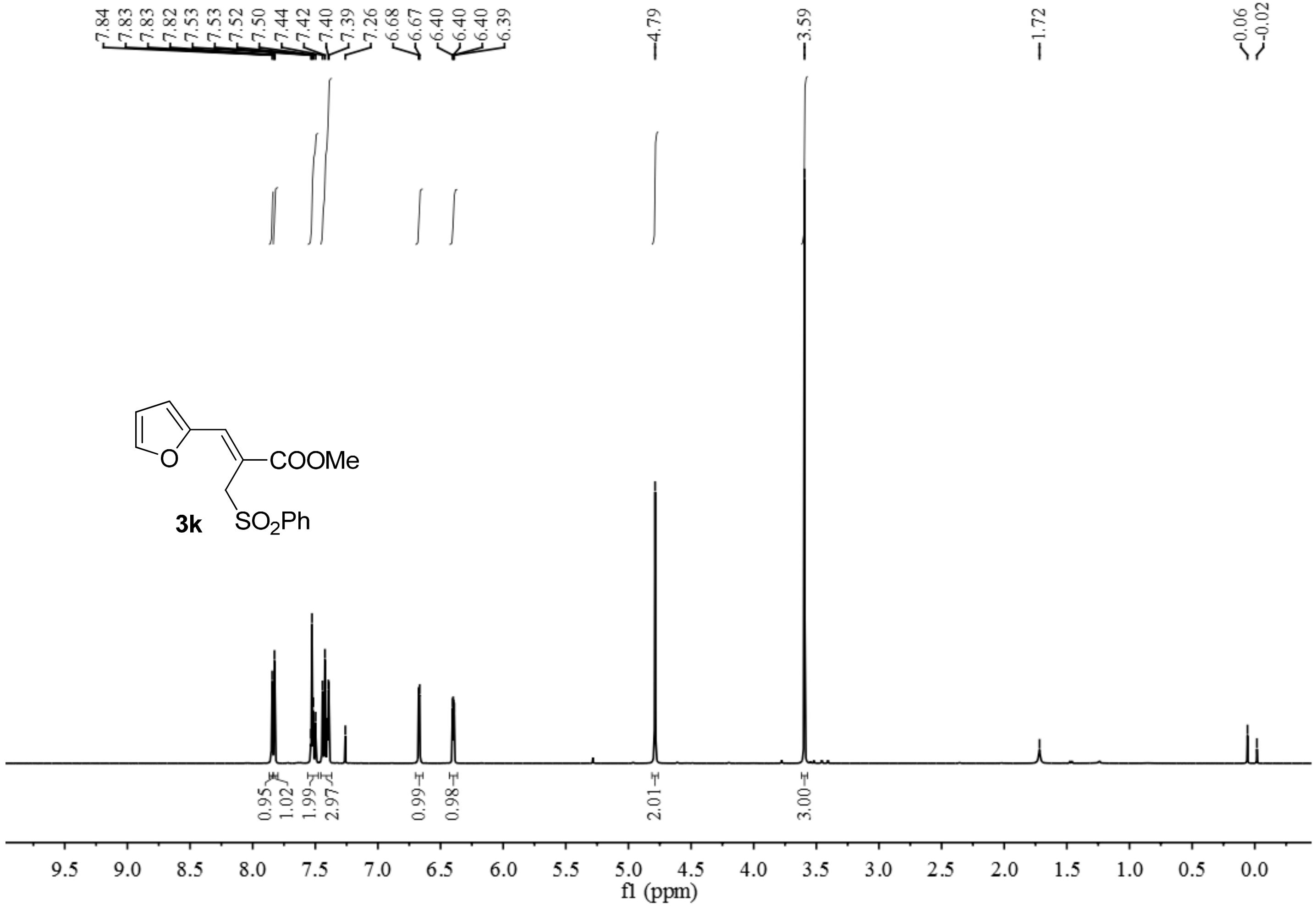

Figure S21. ^{1}H-NMR spectrum of compound **3k**.

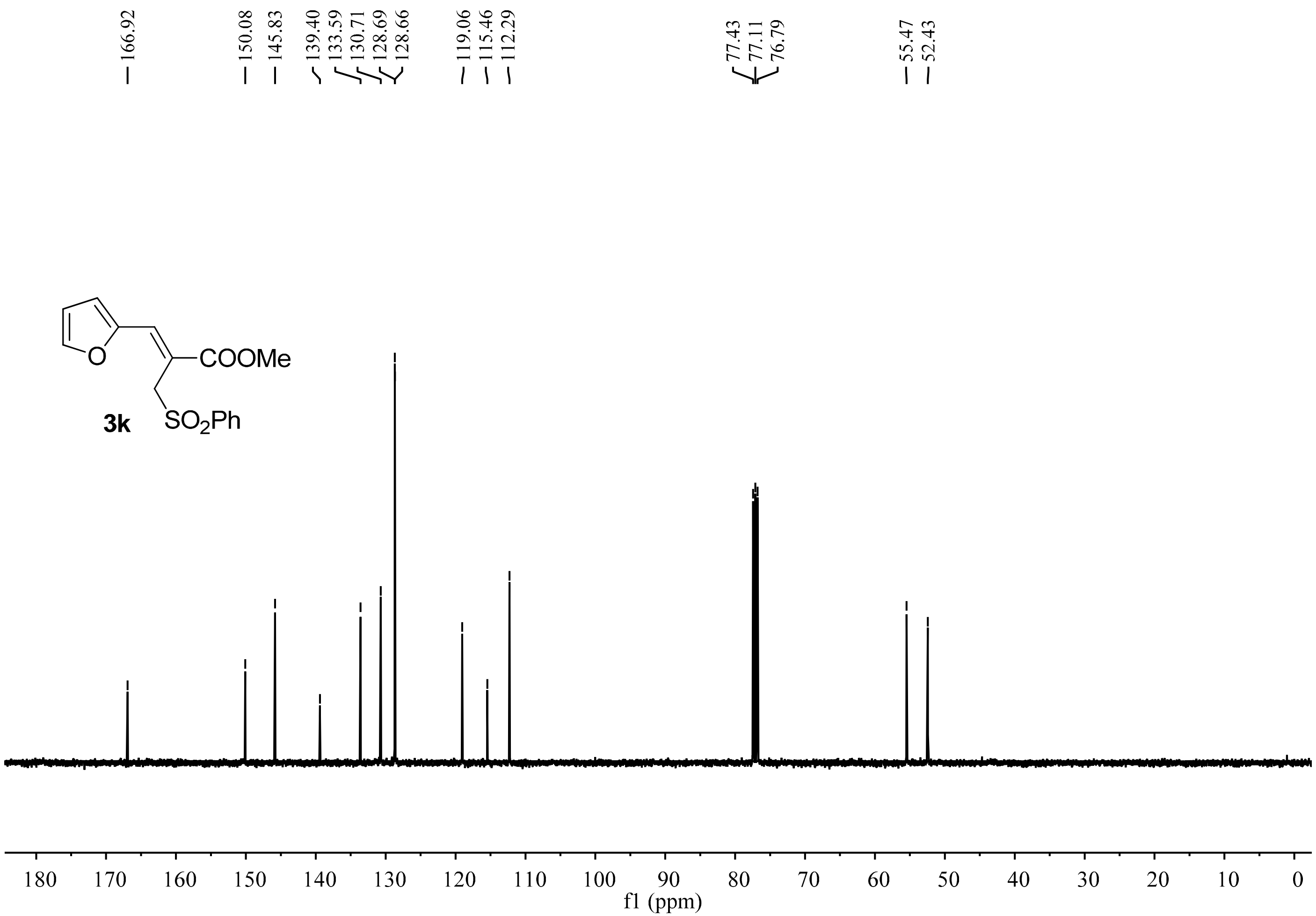

Figure S22. ^{13}C-NMR spectrum of compound **3k**.

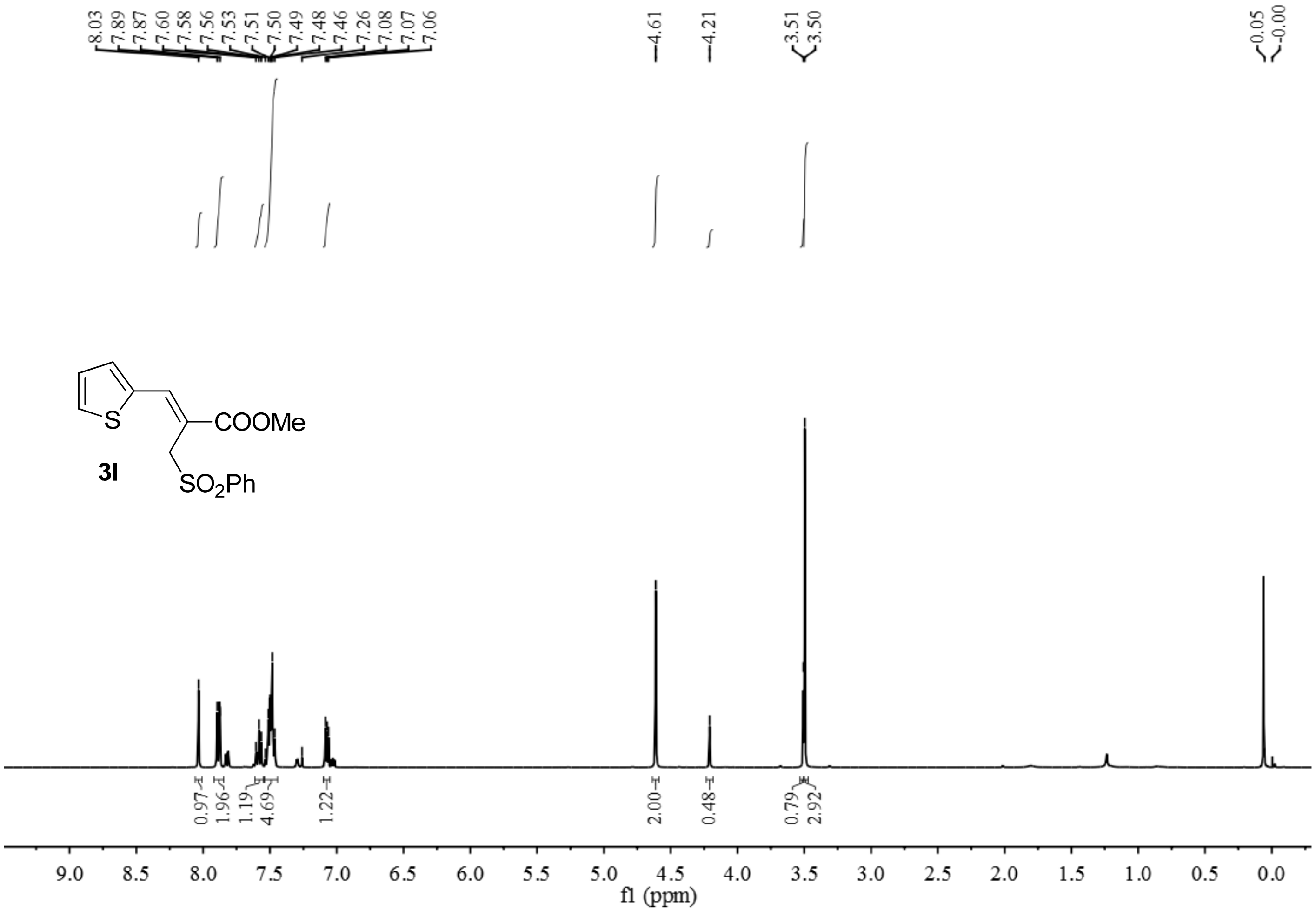

Figure S23. ^{1}H-NMR spectrum of compound **3l**.

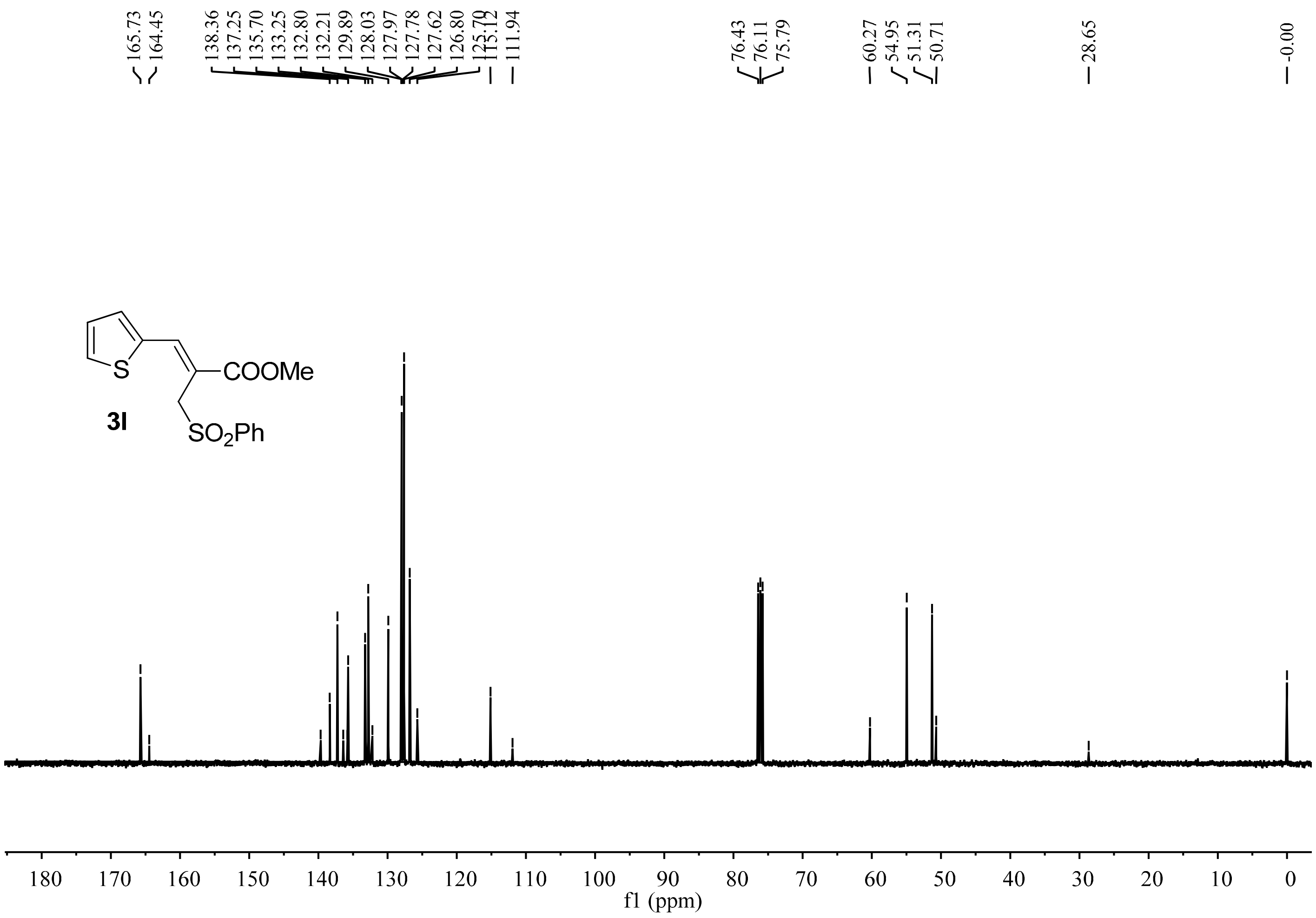

Figure S24. ^{13}C-NMR spectrum of compound **3l**.

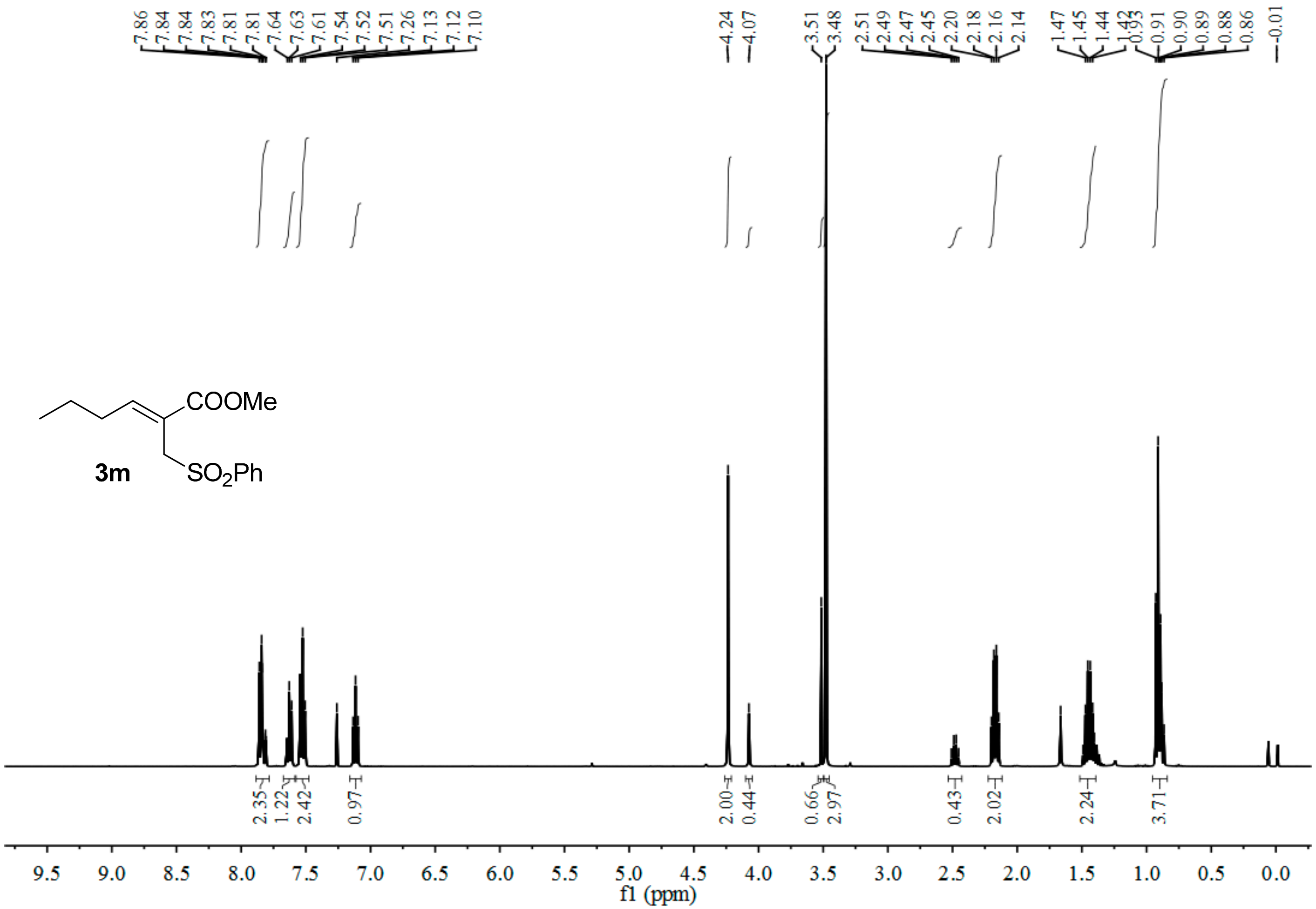

Figure S25. ^{1}H-NMR spectrum of compound **3m**.

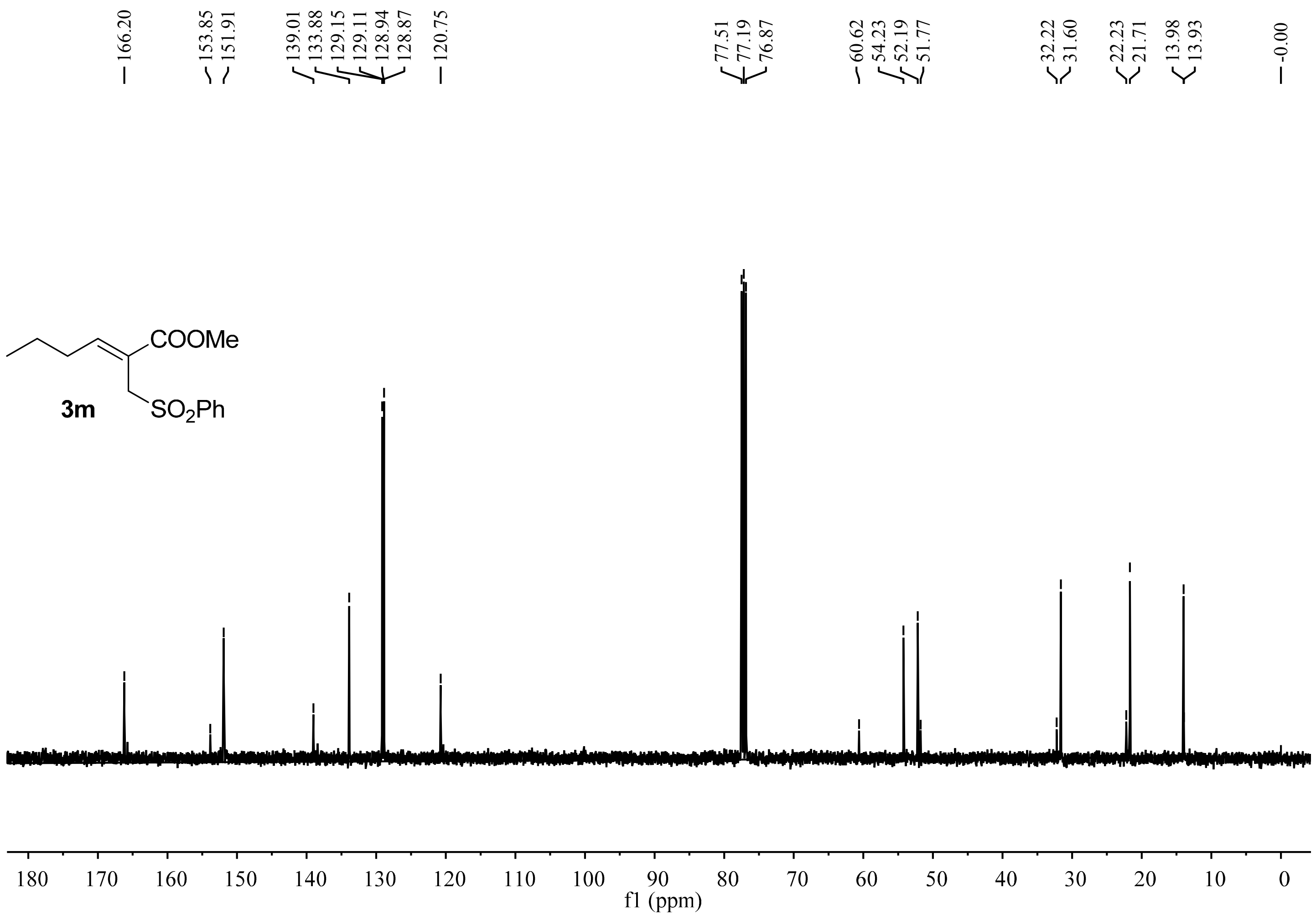

Figure S26. ^{13}C-NMR spectrum of compound **3m**.

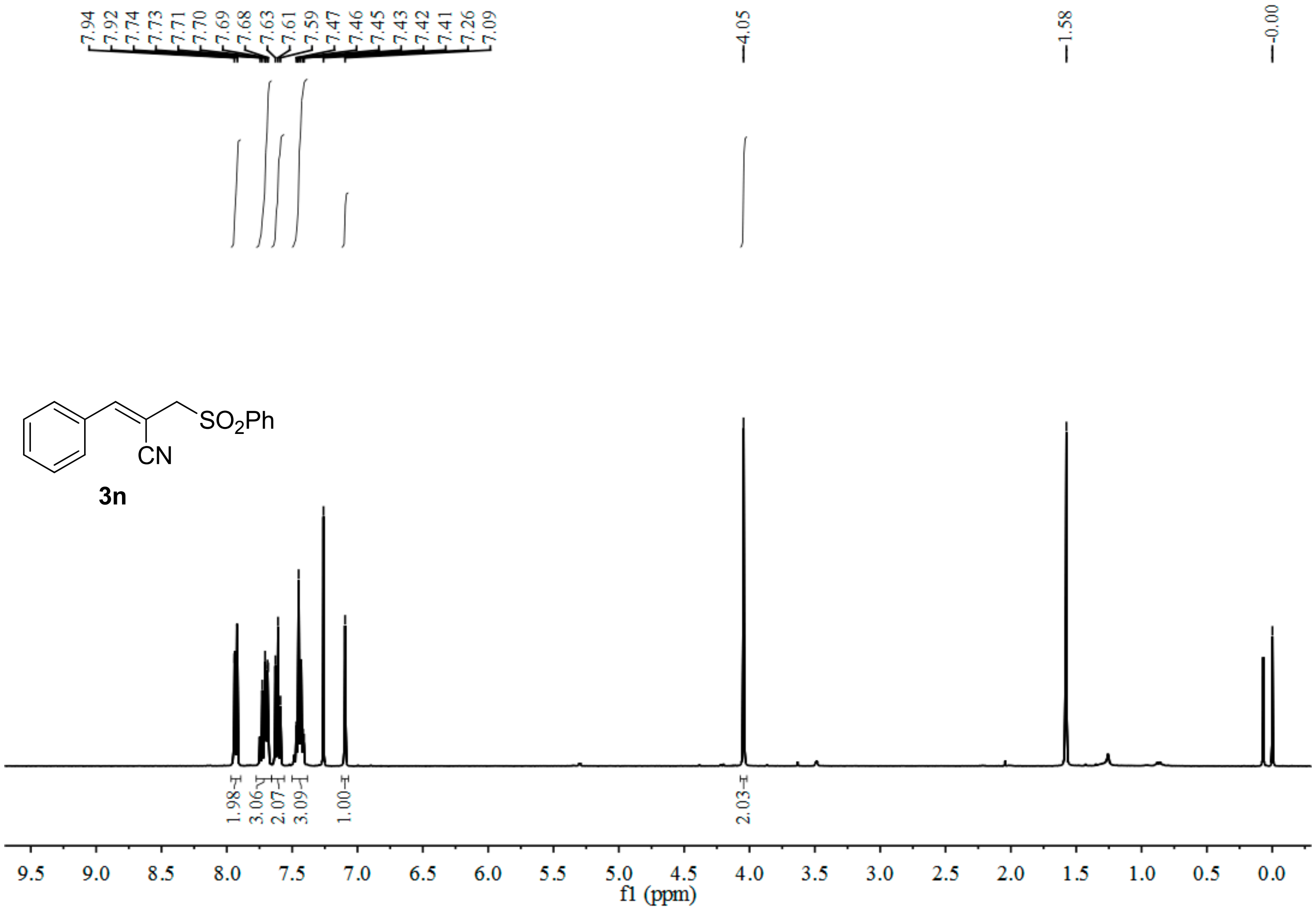

Figure S27. ^{1}H-NMR spectrum of compound **3n**.

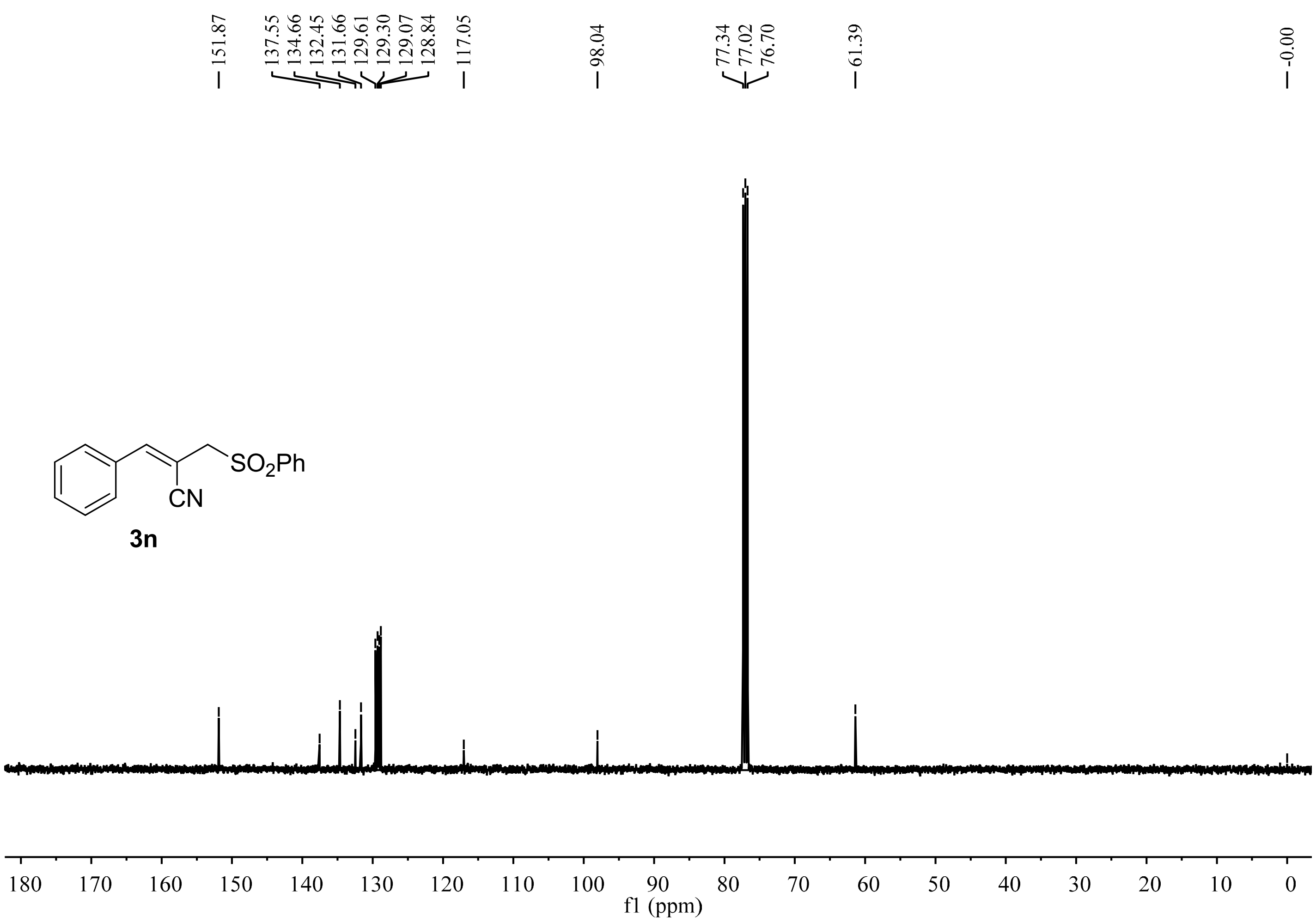

Figure S28. ^{13}C-NMR spectrum of compound **3n**.

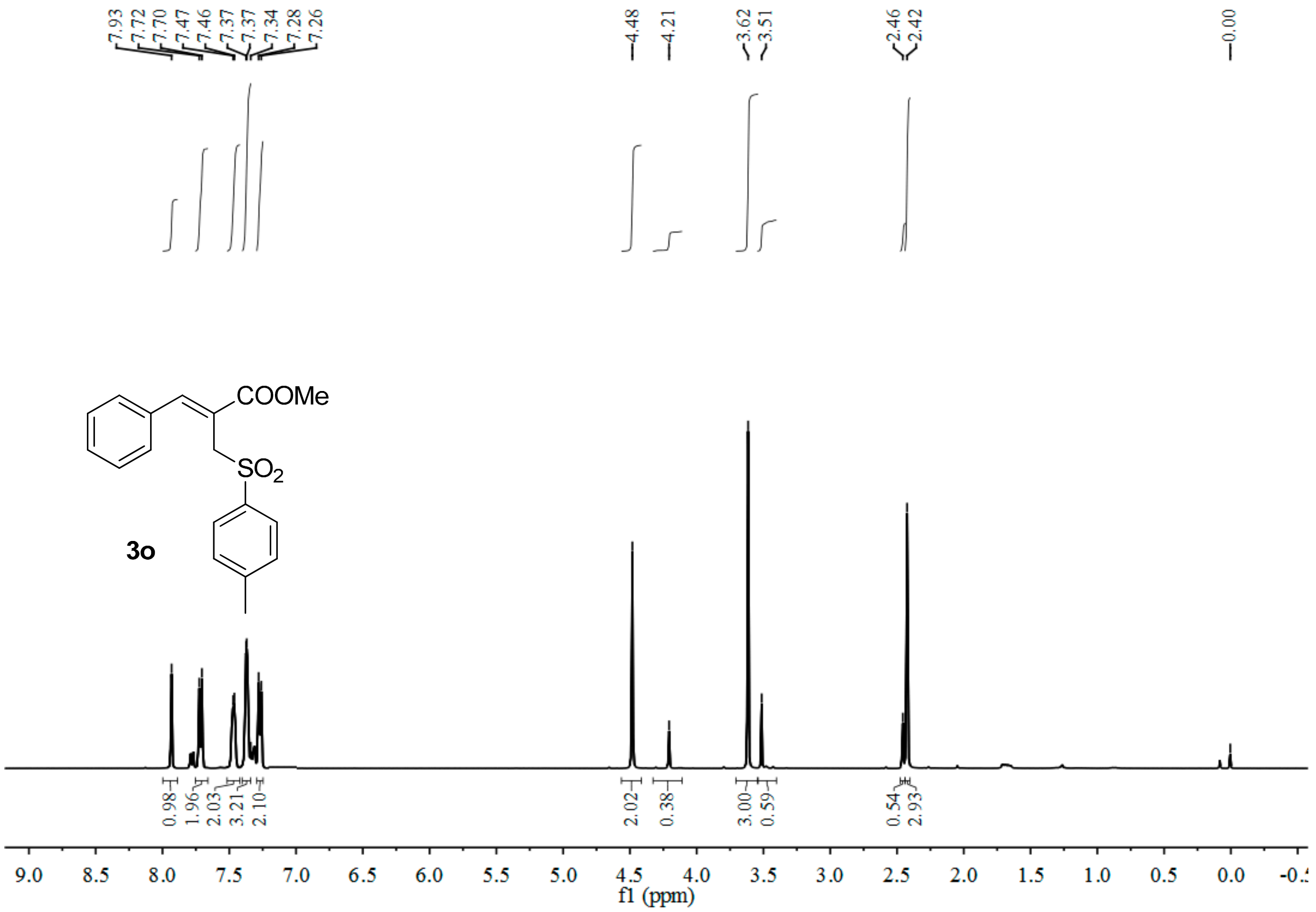

Figure S29. ^{1}H-NMR spectrum of compound **3o**.

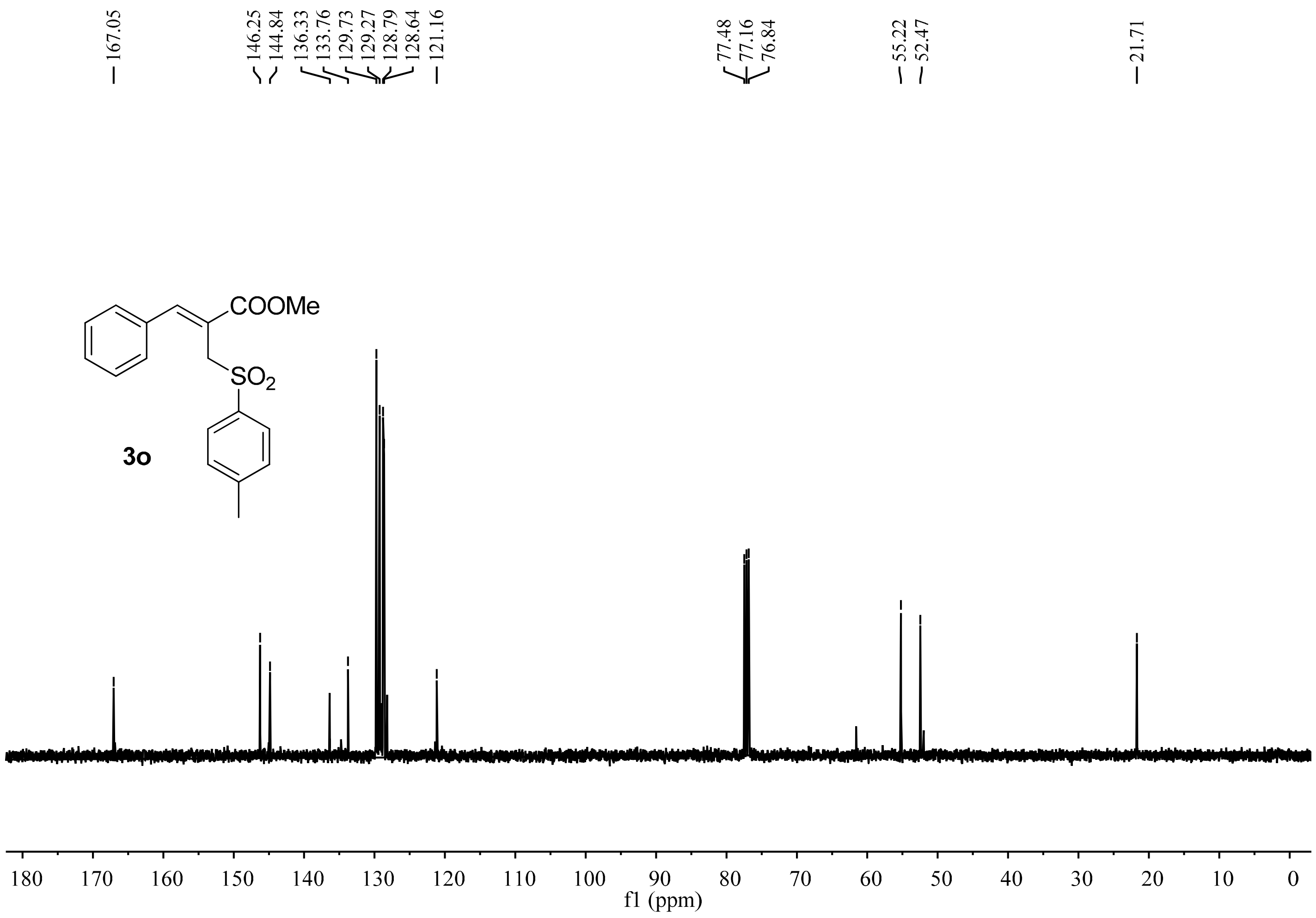

Figure S30. ^{13}C-NMR spectrum of compound **3o**.
